# Physiological and Proteomic Dissection of the Responses of Two Contrasting Wheat Genotypes to Nitrogen Deficiency

**DOI:** 10.3390/ijms21062119

**Published:** 2020-03-19

**Authors:** Mohammad Rezaul Karim, Ruonan Wang, Lu Zheng, Xiaoying Dong, Renfang Shen, Ping Lan

**Affiliations:** 1 State Key Laboratory of Soil and Sustainable Agriculture, Institute of Soil Science, Chinese Academy of Sciences, Nanjing 210008, China; rezaul.karim@issas.ac.cn (M.R.K.); wangruonan@issas.ac.cn (R.W.); luzheng@issas.ac.cn (L.Z.); xydong@issas.ac.cn (X.D.); rfshen@issas.ac.cn (R.S.); 2 University of Chinese Academy of Sciences, Beijing 100049, China

**Keywords:** wheat, nitrogen starvation, leaf chlorophyll, genes, proteome

## Abstract

Nitrogen deficiency usually occurs along with aluminum toxicity in acidic soil, which is one of the major constraints for wheat production worldwide. In order to compare adaptive processes to N deficiency with different Al-tolerant wheat cultivars, we chose Atlas 66 and Scout 66 to comprehensively analyze the physiological responses to N deficiency, coupled with label-free mass spectrometry-based proteomics analysis. Results showed that both cultivars were comparable in most physiological indexes under N deficient conditions. However, the chlorophyll content in Scout 66 was higher than that of Atlas 66 under N deficiency. Further proteomic analysis identified 5592 and 5496 proteins in the leaves of Atlas 66 and Scout 66, respectively, of which 658 and 734 proteins were shown to significantly change in abundance upon N deficiency, respectively. The majority of the differentially expressed proteins were involved in cellular N compound metabolic process, photosynthesis, etc. Moreover, tetrapyrrole synthesis and sulfate assimilation were particularly enriched in Scout 66. Our findings provide evidence towards a better understanding of genotype-dependent responses under N deficiency which could help us to develop N efficient cultivars to various soil types.

## 1. Introduction

Wheat (*Triticum aestivum* L.) is one of the most important staple crops worldwide, providing the majority of calories for about 30% of the world’s population [1], and has considerably higher protein content than any other cereals crop such as maize and rice [2]. In order to maintain or increase yield, nitrogen fertilizer is frequently overused, approximately 100 kg/ha being fed to wheat fields [3]. But the N use efficiency is only 30–40% in wheat production during the first growing season. The overuse of N fertilizers has not only led to low N-use efficiency in wheat fields but has also resulted in severe environmental pollution, especially soil acidification [4,5,6]. Therefore, understanding the mechanisms of wheat response to N deficiency and improving N-use efficiency, such as developing N-efficiency cultivars via molecular breeding, is critical for decreasing the dependence of N fertilizers. It has been reported that, in recent years, approximately 50% of the world’s soil used to cultivate crops has been or is becoming acidic [7]. With the soil being acidic, aluminum toxicity, along with the poor nutrition of N and phosphorus (P), is considered to be the primary factor limiting plant growth in acidic soils, particularly when the soil pH is below 5 [8]. Hence, developing a cultivar which possesses both N efficiency and Al tolerance would be useful to wheat production in acidic soils.

Fortunately, some Al-tolerant wheat cultivars have been developed by breeding programs, and the responses of these cultivars to Al toxicity in acidic soil have been documented [9,10]. In our previous study, unexpectedly, we found that Al-sensitive cultivar Scout 66 performed better under P deficient conditions than that of the Al-tolerant cultivar Atlas 66 [11]. In particular, Scout 66 contained higher chlorophyll content under low P stress than that of Atlas 66. Since it is well known that N is one of the components of chlorophyll, we suspected that Scout 66 can contain high N content or due to the fact of other reasons of N starvation. Indeed, recent studies have shown the complex interplay between N and P [12,13,14]. Similar findings of other studies also suggest that these two most demanded macronutrients for plants must co-exist in internal molecular relationships and it warrants further exploitation. Meanwhile, our previous study also suggested that some potential gene resources or beyond molecular mechanisms of P efficiency as well as probably N efficiency or both could be mined from such kinds of Al-responsive cultivars. Nitrogen shortage in acidic soils is quite normal, so we wondered whether a similar phenomenon (i.e., an Al-sensitive cultivar showing better fitness under N deficiency) exists. However, until now, whether different Al-sensitive cultivars have distinct responses to N deficiency remains limited, and whether the Pi-deficiency responses of Al-sensitive cultivars is similar to N is yet to be revealed.

It is generally accepted that nitrate (NO_3_^−^) is the favored form of N from soil for wheat growth [15,16,17]. It has been reported that there are two classes of NO_3_^−^ transport systems, low-affinity transport systems (LATS), and high-affinity transport systems (HATS), governing NO_3_^−^ uptake in plants, depending on the external NO_3_^−^ concentration [18,19]. It was shown that the *NRT2* gene family, belonging to the HATS, plays a critical role in NO_3_^−^ acquisition from soil under N limitation conditions [20]. However, the temporal transcriptional expression patterns of *NRT1* and *NRT2* gene family and their effects on N uptake in wheat have long been unclear. It is clear, nevertheless, that the NO_3_^−^ transport capabilities of most *NRT2* proteins in plants need the involvement of a chaperone protein, *NAR2*, as part of a two-component high-affinity NO_3_^−^ uptake system [16,21,22]. But, whether this scenario could be applied to wheat remains to be revealed due to the fact of its complex genome. Fortunately, after the recent wheat whole genome sequencing published [23,24], there will be possibilities to understand how wheat responds to NO_3_^−^ deficiency.

With the advances in high-resolution mass spectrometry coupled with the fast and advanced computational algorithms that enable large data sets to be analyzed and displayed in a biologically meaningful way, proteomics have been widely applied to investigate the global protein expression maps of plants, both in model plants and economically important crops including wheat. Increasing evidence has showed that changes at the transcript level are not always consistent with the abundance changes of the encoded proteins, due to the post-translational turnover and alterable translation efficiency, with the modest congruency of the two levels being reported except for high abundance transcripts [25]. Therefore, proteomic analysis, to some extent, has its advantages to uncover some novel aspects in the metabolism of plants subjected to abiotic stresses which could not be revealed by other OMICS approaches. Our previous study has revealed that higher chlorophyll content was observed in Scout 66 under Pi-deficient conditions [11]. In this study, as N content is directly related to chlorophyll content, we were interested to know whether or not Scout 66 may also have better performance under N deficiency.

## 2. Results

### 2.1. Scout 66 Showed Longer Root Length under Nitrogen Deficiency

**Figure 1:**
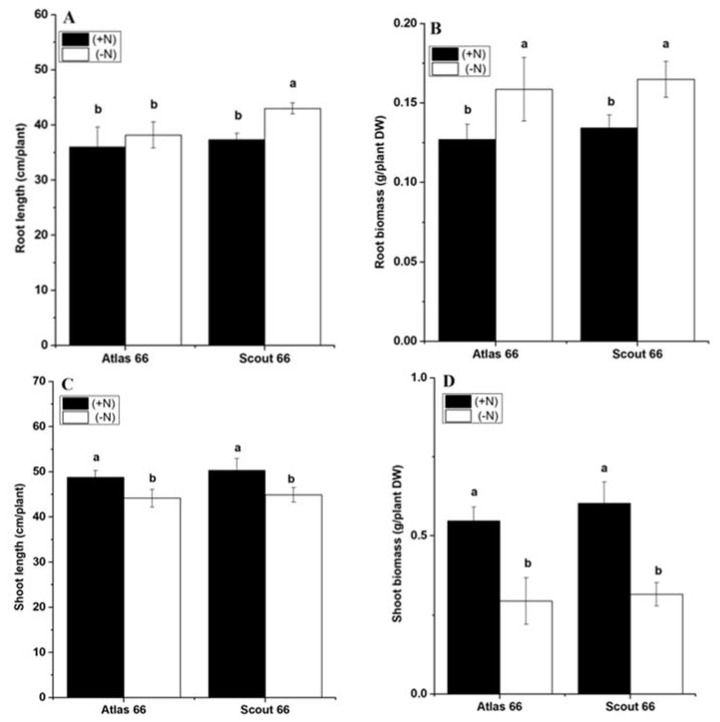
Effect of N deficiency on wheat plant root length (**A**), root biomass (**B**), shoot length (**C**), and shoot biomass (**D**). Wheat plants were transferred from N sufficiency to either N sufficient (2 mM) or deficient (50 µM) conditions for a defined treatment time. Data presented here were sampled at 14 d from both N levels. Bars represent the mean ± SD (*n* = 3). Different small letters indicate statistically significant differences based on LSD tests (*p* ≤ 0.05) among the treatments and cultivars.

Two wheat cultivars, Atlas 66 and Scout 66, with different sensitivities to Al stress, previously reported to respond differently to sole Pi starvation, were used here to investigate the physiological responses to N shortages. Two-week-old seedlings grown in N sufficient solution were transferred to either N sufficient or deficient solution for different treatment time and were sampled at the time point as indicated. Overall, the root lengths of Atlas 66 were comparable under both N deficient and sufficient conditions over the treatment time (Supplementary Materials, Figure S1A). In contrast, N deficiency led to the root lengths significantly increased in Scout 66, 3 days after transfer (Supplementary Materials, Figure S1A). Moreover, under N shortage, the root lengths of Scout 66 were also significantly longer than that of Atlas 66 during long-term treatment (Figure 1A). Consistent with the root length, N deficiency treatment did not lead to the root biomass significantly increasing in both cultivars (Supplementary Materials, Figure S1B), except at 14 d treatment when the root biomass grown under N deficiency was remarkably higher than those grown under N sufficiency, regardless of cultivar, whereas the root biomass did not differ between the two cultivars under the same condition (Figure 1B). Overall, in agreement with the root, both shoot lengths and biomass did not change very much upon N deficiency in both cultivars (Supplementary Materials, Figure S1C,D), except at 14 d of treatment when both shoot lengths and biomass were significantly decreased under N deficiency in comparison with those grown under N sufficiency (Figure 1C,D); whereas there was no significant difference between the two genotypes regardless of N deficiency or sufficiency (Figure 1C,D).Overall it was shown, under long-term N deficiency, that Scout 66 showed longer root lengths than Atlas 66.

### 2.2. Scout 66 Contained Higher Chlorophyll Content Under Nitrogen Deficiency

**Figure 2:**
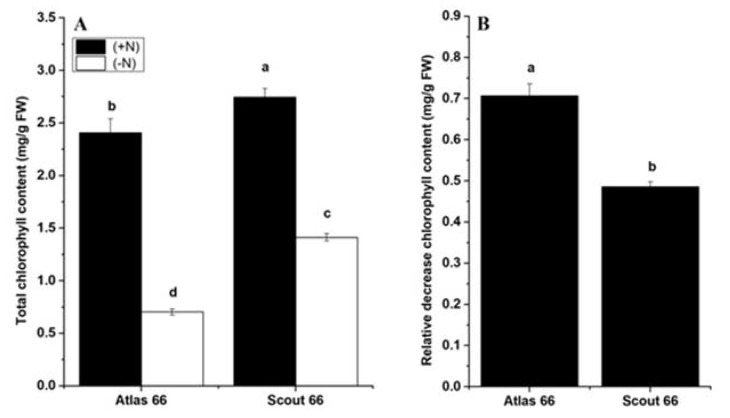
Effect of N deficiency on wheat plant total chlorophyll content (**A**) and the relative decrease of chlorophyll content (**B**). Wheat plants were transferred from N sufficiency to either N sufficient (2 mM) or deficient (50 µM) conditions for a defined treatment time, and the data presented here were sampled at 14 d from both N levels. Bars represent the mean ± SD (*n* = 3). Different small letters indicate statistically significant differences based on LSD tests (*p* ≤ 0.05) among the treatments and cultivars.

We observed that the yellowing colors appeared especially in older leaves at approximately 14 d of N deficiency; therefore, we measured the total chlorophyll contents of the two cultivars from both N levels at this time point. Results showed that the chlorophyll contents significantly decreased when the plants were exposed to N starvation, regardless of the type of cultivar (Figure 2A). However, Scout 66 had substantially higher chlorophyll content than that of Atlas 66, regardless of both N levels, and the chlorophyll content of Atlas 66 was reduced by 70.69% compared with the control, with the reduction amount of 22.14% significantly higher than that of Scout 66 (Figure 2B). In summary, under our conditions, Scout 66 had higher chlorophyll content than that of Atlas 66 under defined treatment time, regardless of the N level.

### 2.3. Scout 66 had Relatively Higher Nitrogen Absorption Efficiency in the Roots While Atlas 66 Showed Slightly Higher Nitrogen Utilization Efficiency in the Roots in Response to Nitrogen Deficiency

**Figure 3:**
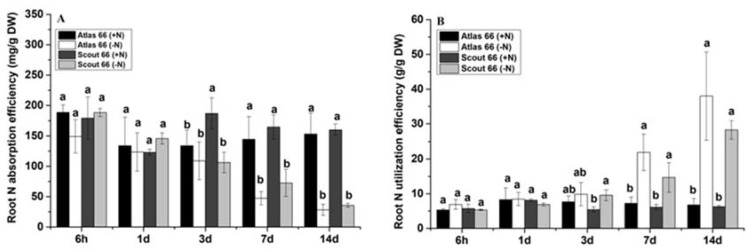
Effect of N deficiency on wheat plant root N absorption efficiency (**A**) and root N utilization efficiency (**B**). Wheat plants were transferred from N sufficiency to either N sufficient (2 mM) or deficient (50 µM) conditions for a defined treatment time, and the data presented here were sampled from a 6 h to 14 d time point from both N levels. Bars represent the mean ± SD (*n* = 3). Different small letters indicate statistically significant differences, based on LSD tests (*p* ≤ 0.05) among the treatments and cultivars.

To understand the N status in root and shoot under N stress, we had calculated N utilization efficiency and the total N accumulation of two wheat cultivars in scheduled treatment time points. As N starvation continued, overall, the root N absorption efficiency was gradually reduced (Figure 3A), but the root N utilization efficiency gradually increased in both cultivars, particularly under long-term treatments (Figure 3B). However, no significant differences in both N absorption and utilization efficiency of the roots were observed before 3d treatment, which is N levels and cultivars independent (Figure 3A,B). It is noticed that, at the 3d treatment point, in Scout 66, N deficiency resulted in significantly lower N absorption efficiency but higher N utilization efficiency than that of the control, respectively; whereas this did not happen in Atlas 66 (Figure 3A,B). In other words, under N deficiency, in the roots, the N absorption efficiency of Scout 66 decreased more than that of Atlas 66, but the N utilization efficiency increased more than that of Atlas 66. Thus, it is suggested that, upon N deficiency, Scout 66 roots might have higher N utilization efficiency, while Atlas 66 roots might have higher N absorption efficiency. Under long-term N deficiency treatments from 7 d to 14 d, the root absorption efficiency of both cultivars gradually decreased 54.42–80.84% in comparison with the control, while the N utilization efficiency of roots kept increasing, 147.05–485.7% in comparison with the control of which the N utilization efficiency of Atlas 66 roots was shown to be 62.9% and 134.27% higher than that of Scout 66 at the treatment time points of 7 d and 14 d, respectively, whereas the N absorption efficiency of Scout 66 was shown to be 11.86% and 3.12% higher than that of Atlas 66 under N deficient conditions at the same treatment time points. In conclusion, with the time increase of N starvation, in the roots, Atlas 66 showed relatively higher N utilization efficiency, while Scout 66 had slightly higher N absorption efficiency.

### 2.4. Scout 66 and Atlas 66 Showed Different Nitrogen Contents Both in the Roots and the Shoots

**Figure 4:**
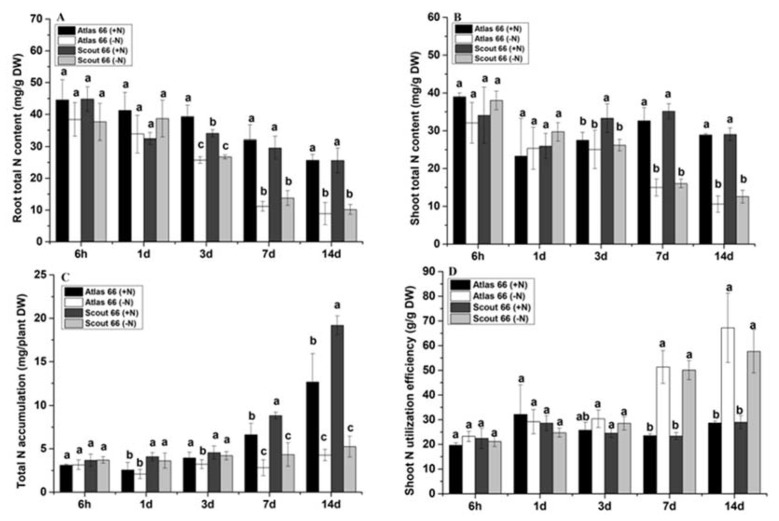
Effect of N deficiency on wheat plant root total N content (**A**), shoot total N content (**B**), total N accumulation (**C**), and shoot N utilization efficiency (**D**). Wheat plants were transferred from N sufficiency to either N sufficient (2 mM) or deficient (50 µM) conditions for a defined treatment time, and the data presented here were sampled from a 6 h to 14 d time point from both N levels. Bars represent the mean ± SD (*n* = 3). Different small letters indicate statistically significant differences based on LSD tests (*p* ≤ 0.05) among the treatments and cultivars.

Given that there are differences in root N absorption and utilization efficiency in the two cultivars, we wanted to know whether the N contents and accumulation in these cultivars were different. As N starvation continued, in general, the N contents in both roots and shoots were gradually reduced under N deficiency, regardless of cultivar (Figure 4A,B). However, before 3 d of treatments, there was no significant difference in the N contents of both roots and shoots, regardless of cultivars and N levels (Figure 4A,B). After 3 d, at the same N levels, there was no significant difference in N contents of both shoots and roots in both cultivars. Although there was no significant difference in N contents of both roots and shoots under low N stress at 3 d treatment, the total N content of Atlas 66 in the roots was significantly higher than that of Scout 66 (Figure 4A), while the total N content of Atlas 66 in the shoots was significantly lower than that of Scout 66 under N sufficient conditions (Figure 4B). Accordingly, the total N content of Atlas 66 in the roots decreased by 34.49% in comparison with the control, with the reduction amount being 13.07% higher than that in Scout 66; while the total N content of Atlas 66 in the shoots decreased by 8.84% in comparison with the control, with the reduction amount being 12.61% lower than that in Scout 66 under N starvation (Figure 4A,B). Overall, the conclusion is that the relative total N content of Scout 66 in the roots was high, while that of Atlas 66 in the shoots was high.

### 2.5. Scout 66 Accumulated More Nitrogen Than Atlas 66 Did

Overall, over time, the accumulation of total N showed a trend of gradual increase under N sufficient conditions, while the change trend was not obvious under low N stress (Figure 4C). Before 3 d of treatments, there was no significant difference in the accumulation of total N between two N levels in both cultivars. From 3 d onwards, the total N accumulation of Atlas 66 was decreased by 18.21–66.21% compared with the control, and the reduction trend was gradually increased over time. While in Scout 66, the decreasing trend of total N accumulation was much delayed and the trend was observed to be gradually increased at 7 d and beyond upon N deficiency, with the decreases of total N accumulation being 50.94% and 72.63%, respectively at 7 d and 14 d, compared with the control. It is noticed that at the 14 d of N deficiency, the reduction amount in Scout 66 was 6.42% higher than that of in Atlas 66.

Although, overall, Atlas 66 and Scout 66 had comparable total N contents in the shoots in both N levels (Figure 4B), the chlorophyll content of Scout 66 was significantly higher than that of Atlas 66 under low N stress (Figure 2), so we wondered whether or not Scout 66 possessed a higher N utilization efficiency in the shoots. As shown in Figure 4D, there was no significant difference in N utilization efficiency between the two cultivars at different N levels within 3 d treatments. After 3 d, there was still no significant difference between the two cultivars at the same N level. But compared to the control, both cultivars had significantly higher N utilization efficiency in the shoots. At 7 d and 14 d, compared to the control, the shoot N utilization efficiency of Atlas 66 increased by 114.32% and 101.72%, while the N utilization efficiency of Scout 66 increased by 117.63% and 135.32%, respectively. In summary, under low N stress, the shoots of Scout 66 had relatively higher N utilization efficiency.

### 2.6. The Changes in Nitrate Concentrations of Two Wheat Cultivars in Response to Nitrogen Deficiency

**Figure 5:**
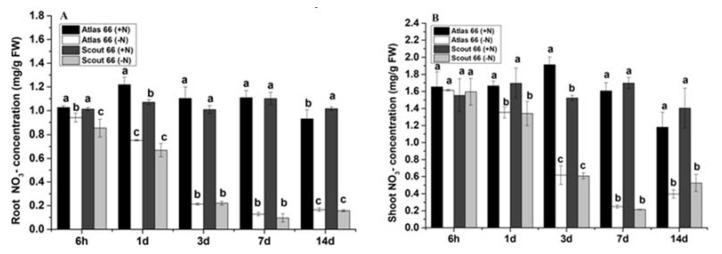
Effect of N deficiency on wheat plant root NO_3_^−^ concentration (**A**) and shoot NO_3_^−^ concentration (**B**). Wheat plants were transferred from N sufficiency to either N sufficient (2 mM) or deficient (50 µM) conditions for a defined treatment time; data presented here were sampled from 6 h to 14 d time points from both N levels. Bars represent the mean ± SD (*n* = 3). Different small letters indicate statistically significant differences based on LSD tests (*p* ≤ 0.05) among the treatments and cultivars.

Since NO_3_^−^ from soil is the preferred form of N for wheat growth, to take insight into the responses of wheat to N stress, we thus further measured both root and shoot NO_3_^−^ concentrations. In general, over the period of N starvation, the NO_3_^−^ concentrations both in the roots and shoots showed a significantly decreasing trend in comparison with that under N sufficiency, and the responses of roots to N deficiency were more sensitive than that of shoots in both cultivars (Figure 5). Surprisingly, the root NO_3_^−^ concentrations were shown reduced significantly at the early treatment (6 h of N stress) time point in both cultivars, whereas it occurred at 1 d of treatment in the shoots (Figure 5). It was noticed that under N deficiency, Atlas 66 had significantly higher NO_3_^−^ concentration in the roots than that of Scout 66 in the early treatment (Figure 5A). Under short-to-mid-term treatments (at 1 d and 3 d), the NO_3_^−^ concentrations of Atlas 66 had a greater reduction than that of Scout 66; while under long-term treatments, the reduction of NO_3_^−^ concentrations in Atlas 66 was gradually lower than that of Scout 66, by 2.82% (7 d) and 2.39% (14 d) lower than that of Scout 66, respectively (Figure 5A). The results showed that the relative amount of NO_3_^−^ concentrations in the roots of Atlas 66 was higher than that of Scout 66 when the time was increased.

As mentioned above, overall, with the treatment time expansion, the shoot NO_3_^−^ concentrations were decreased under N deficiency in both cultivars, and a significant reduction started from 1 d of N starvation (Figure 5B). At the 3 d treatment time point, when N supply was sufficient, Scout 66 had a significantly lower NO_3_^−^ concentration than that of Atlas 66. At other treatment time points, however, regardless of N levels, the shoot NO_3_^−^ concentrations did not show any significant difference between the two cultivars, though it seems that, at early time points, Atlas 66 contained relatively higher NO_3_^−^ concentrations, but at long-term treatments (14 d), Scout 66 had relatively higher NO_3_^−^ concentration under N deficiency.

### 2.7. Differential Expression of Marker Genes was Induced by Nitrogen Deficiency

**Figure 6:**
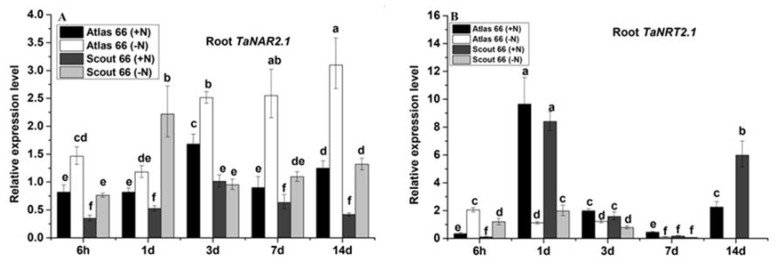

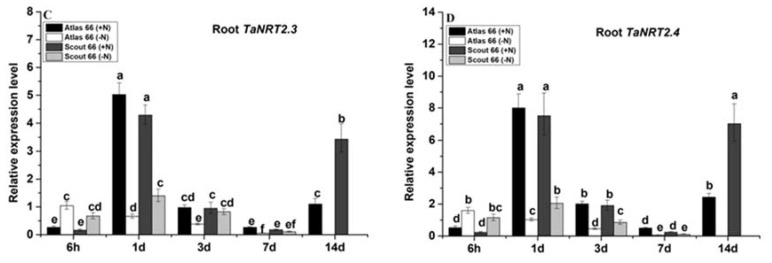
The relative expression level of four targeted genes, *TaNAR2.1* (**A**), *TaNRT2.1* (**B**), *TaNRT2.3*, (**C**), *TaNRT2.4* (**D**) in roots of Atlas 66 and Scout 66 grown in N sufficient (2 mM) and deficient (50 µM) conditions from 6 h to 14 d time points from both N levels. Bars represent the mean ± SD (*n* = 3). Different small letters indicate statistically significant differences based on LSD tests (*p* ≤ 0.05) among the treatments and cultivars.

It has been known and well established that plants have evolved and altered different NO_3_^−^ transporters to cope with N stress [15,26]. To understand the molecular responses of the two wheat cultivars to N deficiency, we chose to determine the expression of the N-responsive marker genes, the high affinity NO_3_^−^ transporters along with the functional partner, in different scheduled time points by using quantitative RT-PCR (*qRT-PCR*). Expression of the two component high-affinity uptake system, *NAR2.1*, was overall significantly induced by N deficiency in both cultivars, and the induction was greater in Atlas 66 than in Scout 66 under long-term N starvation (Figure 6A). Expression of three NO_3_^−^ transporters (*TaNRT2.1, TaNRT2.3,* and *TaNRT2.4*) of the *TaNRT2* gene family was analyzed, and the results showed that the expression of all the transporter genes tested in this study were dramatically decreased in both cultivars over the N starvation time, except at 6 h of N stress (Figure 6B–D). Interestingly, at the treatment time point of 6 h, expression of these genes was significantly induced in both cultivars and decreased gradually from 1 d of N stress and onwards. It was noticed that, at 14 d of the time point, the extent of expression of these genes was more significant in Scout 66 than that of in Atlas 66 when the N supply was sufficient (Figure 6B–D).

### 2.8. Protein Identification and Differentially Accumulated Proteins in the Two Cultivars Under Nitrogen Starvation

**Figure 7:**
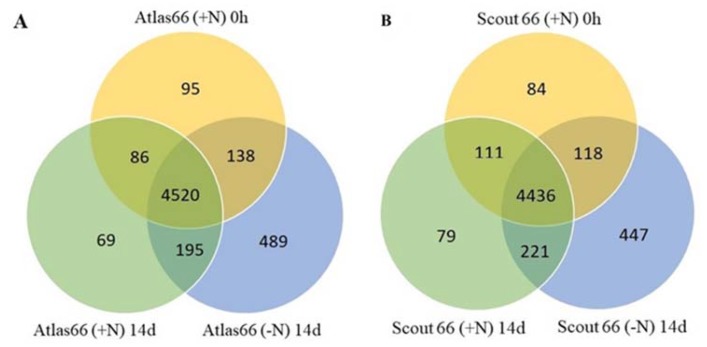
Venn diagram of total proteins identified in the leaves of Atlas 66 and Scout 66 in this study. The Venn diagram showed the total number of proteins identified among three conditions in both Atlas 66 (**A**) and Scout 66 (**B**).

**Figure 8:**
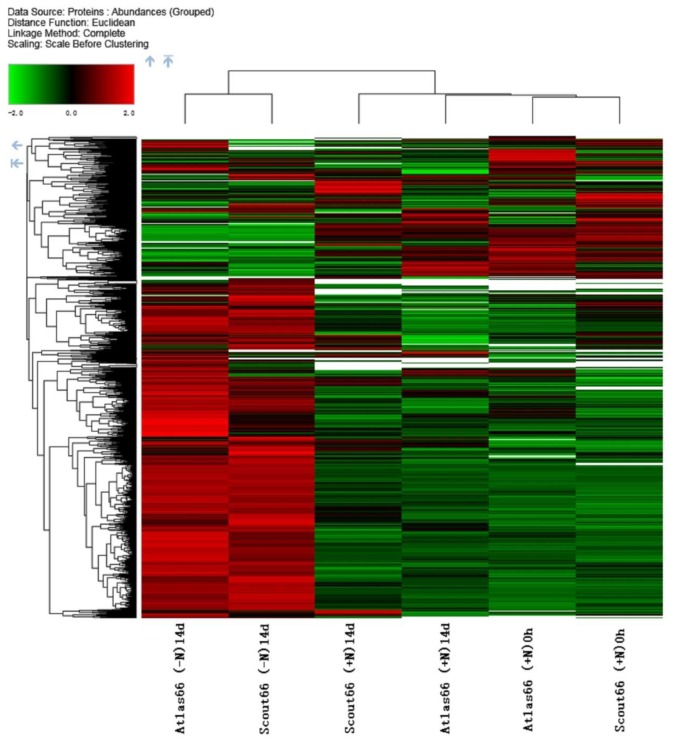
The hierarchical cluster analysis of protein abundance among sample groups in leaves of Atlas 66 and Scout 66. Columns represent the average of each treatment combination and rows indicate abundance of proteins. The relative abundance of proteins is indicated from low to high, marked as from green to red.

To further understand the molecular mechanism of N deficiency responses of the two wheat cultivars, the leaf total proteins were extracted and changes of proteome composition upon N deficiency explored using a label-free quantitative proteomic technique. Since the main difference between the two cultivars upon N starvation was the leaf chlorophyll content at 14 d of N stress; therefore, proteomic analyses in this time were focused on the changes of leaf protein profiling between N sufficient and deficient conditions in both cultivars. The quality and quantity of proteins extracted from leaf samples before treatment or after 14 d treatment of either N sufficiency or deficiency were assayed before going to next steps. From the SDS-PAGE gel, it showed that similar protein patterns were obtained after protein separation, indicating that the reproducibility of both protein extraction and concentration determination was high in different samples from three biological repeats (Supplementary Materials, Figure S2). Subsequent proteomic analysis identified a total of 4839, 4870, and 5342 proteins in the Atlas 66 samples of 0 h and 14 d of N sufficient and deficient treatments, respectively. Merging all the data obtained from the three treatments led to a total of 5592 proteins identified in Atlas 66, with an overlap of 4520 proteins (Figure 7A). A total of 4749, 4847, and 5222 proteins were identified in the Scout 66 samples of 0 h, 14 d of N sufficient and deficient treatments, respectively, by similar analysis, and a total of 5496 proteins were obtained after merging all the data from three treatments, with an overlap of 4436 proteins (Figure 7B). It is noticed that, in both cultivars, a notably higher amount of proteins were identified under N deficient conditions than that under N sufficiency. Hierarchical visualized expression patterns of different treatments were further performed, and the cluster relationships confirmed our treatments and analyses were reliable (Figure 8). This analysis also suggested changes in protein profiles mediated by N deficiency were more significant than that by development (0 h versus 14 d) regardless of the cultivar (Figure 8).

A cutoff of fold change ≥ 2 and adjusted *p*-value ˂ 0.05 was set to mine the differentially accumulated protein (DAP) among treatments or cultivars. After merging the data obtained from N sufficiency and deficiency at a 14 d treatment time point, a total of 5497 and 5412 proteins were identified in Atlas 66 and Scout 66 (Supplementary Materials, Figure S3A,B), respectively, with a subset of 658 (Supplementary Materials, Table S1) and 734 proteins (Supplementary Materials, Table S2) being differentially accumulated. Four hundred and fifty-one and 474 of which increased in abundance in Atlas 66 and Scout 66 (Supplementary Materials, Figure S4A,B), whereas 207 and 260 of which decreased in abundance in Atlas 66 and Scout 66 (Supplementary Materials, Figure S4A,B), respectively. In addition, several hundreds of DAPs were found among cultivars under the same conditions. A subset of 606 (Supplementary Materials, Table S3), 621 (Supplementary Materials, Table S4), and 410 (Supplementary Materials, Table S5) proteins out of 5067, 5107, and 5539 proteins (Supplementary Materials, Figure S3C–E), obtained from 0 h and 14 d of N sufficiency and deficiency in both cultivars were shown to be differentially accumulated in abundance, respectively. Of which, the abundance of 271 proteins at 0h increased, the abundance of 275 and 143 proteins at 14 d increased in N sufficiency and N deficiency; whereas the abundance of 335 proteins at 0 h decreased, and the abundance of 346 and 267 proteins decreased in N sufficiency and deficiency at 14d (Supplementary Materials, Figure S4C–E), respectively.

### 2.9. Differentially Accumulated Proteins Reveal New Aspects of the Nitrogen Deficiency Responses

**Figure 9:**
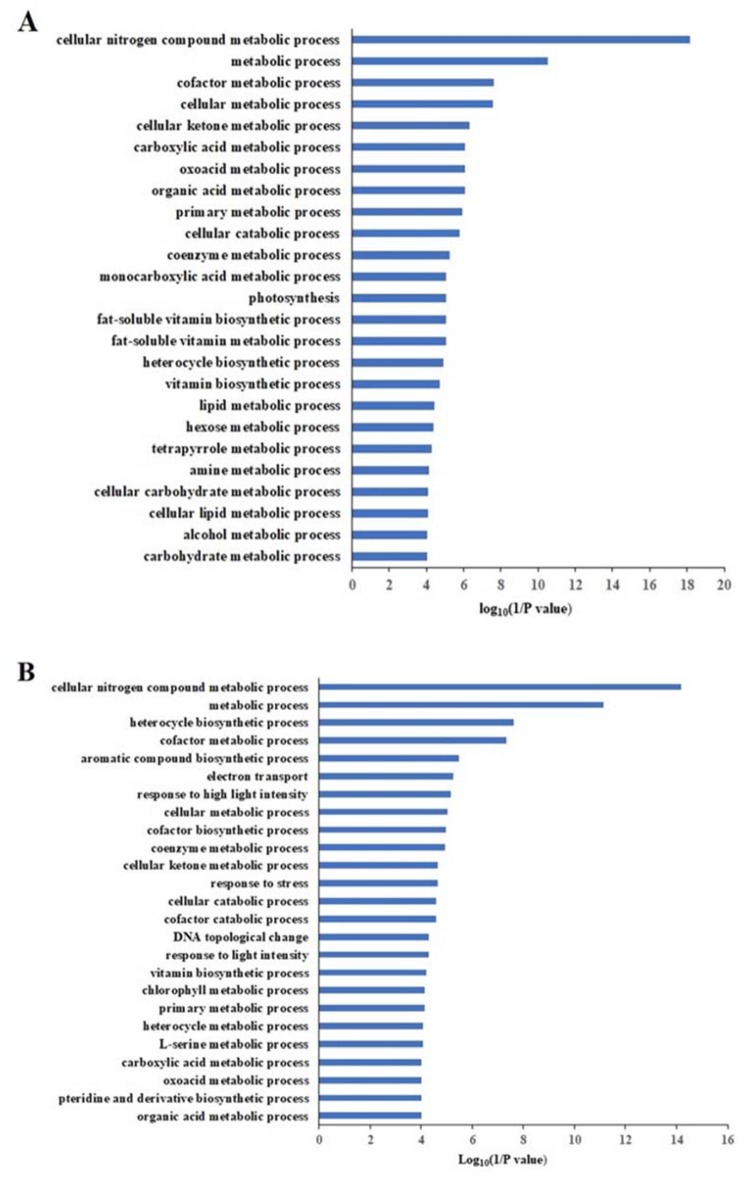
The Gene Ontology (GO) enrichment analysis of differential accumulated proteins (DAPs) in primary functional categorization of Atlas 66 (**A**) and Scout 66 (**B**) (biological process, *p* < 0.01).

Gene ontology (GO) analysis was performed to obtain an overview of the biological processes under N deficiency. First, the analysis of all proteins identified in Scout 66 and Atlas 66 resulted in GO terms that were too complex (shown in the Figure 9); therefore, the results are not presented in this paper. We then focused our GO analysis only on the DAPs and mainly presented the GO categories of biological processes, since the GO categories of cellular components and molecular functions could provide less useful information in our study (but detailed information on them can be found in the detailed GO data sets provided in the Supplementary Materials, Tables S6,S7). The GO analysis of 658 DAPs in Atlas 66 between N deficiency and sufficiency resulted in the biological process of metabolic process was the most enriched (Supplementary Materials, Figure S5A), with the detailed GO data information being shown in the Supplementary Materials, Table S6, of which the biological processes of cellular nitrogen compound metabolic process was the most enriched. Other enriched processes included primary metabolic process, photosynthesis, etc, with 25 of the most enriched processes (*p* < 0.0001) shown in Figure 9A. Overall, the biological process of the 734 DAPs from Scout 66 was similar to that of Atlas 66, with the cellular nitrogen compound metabolic process being the most enriched (Supplementary Materials, Figure S5B). Nevertheless, some Scout 66 specific processes were observed in the top 25 GO terms such as aromatic compound biosynthetic process, response to high light intensity, etc. (Figure 9B), with the detailed GO information of DAPs from Scout 66 being found in Supplementary Materials, Table S7.

### 2.10. Differentially Enriched Pathways Between Atlas 66 and Scout 66 Under N Deficiency

**Figure 10:**
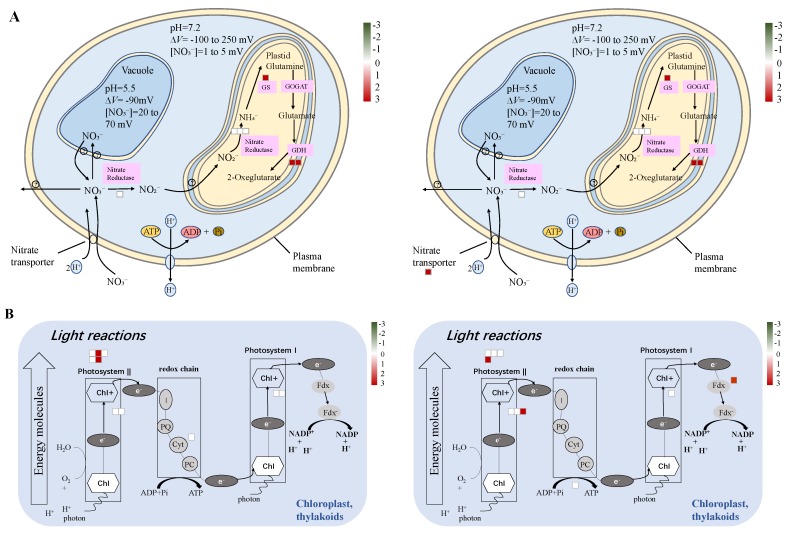
Overviews of NO_3_^−^ metabolism (**A**) and light reactions (**B**) of the DAPs. Both in the NO_3_^−^ metabolism and light reactions. From left to right are Atlas 66 and Scout 66, and all of them are analyzed in and exported from MapMan software and the image quality was further improved accordingly.

**Figure 11:**
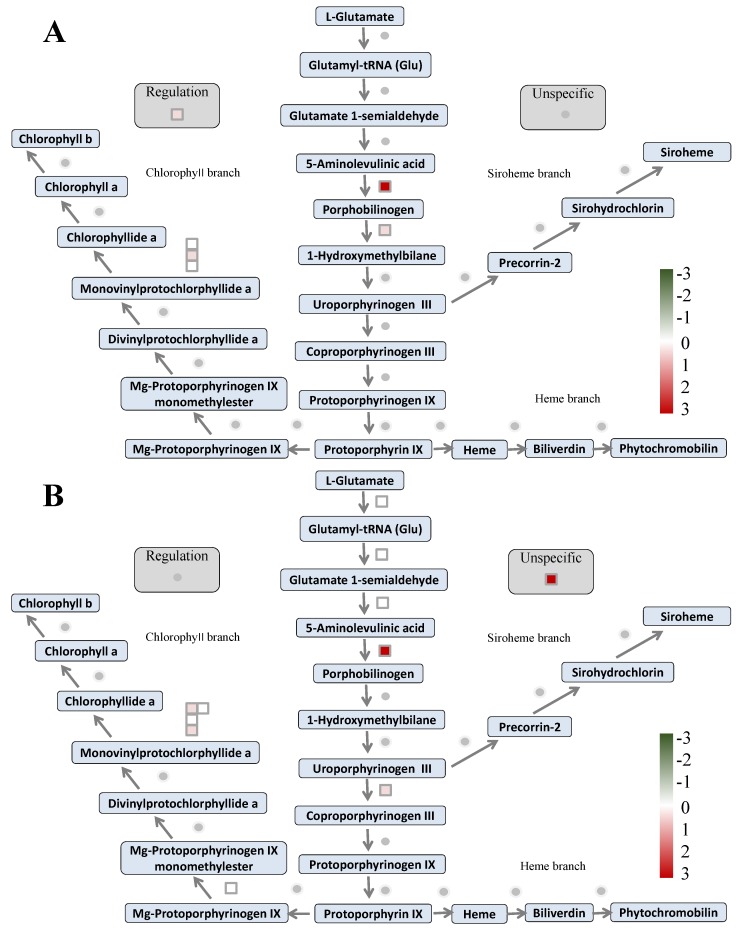

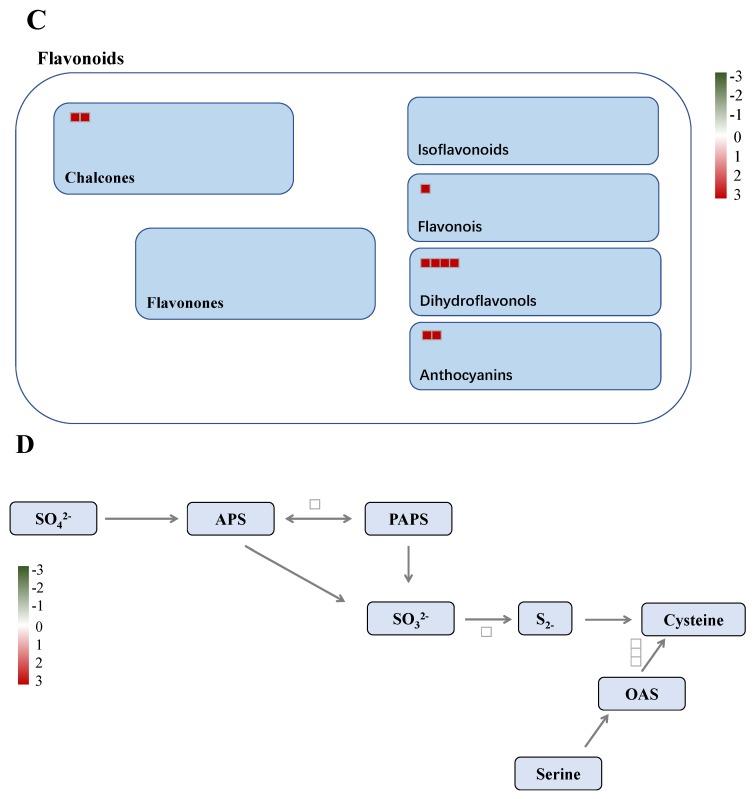
Overviews of tetrapyrrole synthesis (**A**,**B**), flavonoids metabolism (**C**), and sulfate metabolism (**D**) of the DAPs. Panels (**A**) and (**B**) indicate tetrapyrrole synthesis enriched in Atlas 66 and Scout 66, respectively; Panels (**C**) and (**D**) represent the enriched pathways of flavonoids metabolism in Atlas 66 and of sulfate metabolism in Scout 66; all of them were analyzed in and exported from MapMan software and the image quality was further improved accordingly.

In order to further analyze the metabolic pathway of N deficiency responses of two wheat cultivars, we performed MapMan analysis of the DAPs. In agreement with the results of the GO analysis, overall, the MapMan analysis showed that most metabolic pathways were similar in both cultivars upon N deficiency (Supplementary Materials, Figure S6A,B), with N metabolism (Figure 10A) and light reactions (Figure 10B) being enriched. Although the pathway of tetrapyrrole synthesis was also enriched in both cultivars, it was the most enriched in Scout 66 (Figure 11B), with 11 DAPs being presented, while only being relatively enriched in Atlas 66 (*p* = 0.0782) with 6 DAPs observed (Figure 11A). The DAPs involved in the tetrapyrrole synthesis were listed in Table 1. Other relatively enriched pathways common in both cultivars was TCA (tricarboxylic acid cycle)/org (organic matter) transformation (Supplementary Materials, Figure S6A,B). Interestingly, the DAPs involved in N metabolism showed a similarly changing trend in protein abundance in both cultivars (Figure 10A). It should be noticed, however, that an increase of a NO_3_^−^ transporter, Traescs5b02g414000.3, was only observed in Scout 66 upon N deficiency, with it being de novo induced (fold change in abundance = 100) as shown in Table 1 and Figure 10A. MapMan analyses also revealed some differentially enriched pathways between Atlas 66 and Scout 66 under N deficiency. In Atlas 66, secondary metabolism, especially flavonoids metabolism was specifically enriched, with 8 DAPs being observed to be involved in various flavonoids metabolism (Figure 11C and Table 1), while sulfate assimilation was particularly enriched in Scout 66 (Figure 11D and Table 1).

## 3. Discussion

Over past 50 years, N fertilizers have undoubtedly been an efficient way to increase crop production. As one of the main staple food crops, wheat production, to some extent, is tightly correlated to the N status in soil [27], particularly the preferred N form NO_3_^−^. In order to meet the demand of production for the ever-increasing world population, over wheat’s demand, N fertilizers are often applied into the soil all around the world. Although this practice can maintain or increase wheat yield, it leads also to the decrease of N use efficiency and results in major detrimental effects on the environment, including soil acidification. Soil acidification is not only directly related to the well-known toxicity of Al, but also directly associated with the reduction of utilization efficiency of important mineral elements such as N and P [28].

Similarly, in order to exclude the side effects of Al toxicity and at the same time the interaction of Al and N is not the focus, we therefore cultured the wheat seedlings in a controlled hydroponic system and the sole variable is the nitrate concentrations, as indicated in materials and methods.

Overall, under N deficiency, most physiological traits measured did not show sharply different between two cultivars over the treatment time (Figure 1), which is consistent with our previous study [11], suggesting that the responses of the two cultivars to N deficiency generally are similar in physiology in defined time course. However, our finding revealed that Scout 66 contained higher chlorophyll content under both N sufficiency and deficiency than that of Atlas 66 (Figure 2), suggesting that Scout 66 could contain higher N content or higher N efficiency. N is the component of chlorophyll, and it is well established that N shortage will result in the decrease of chlorophyll content, finally leading to leaf yellowing. So, we first compared the total N contents between two cultivars under both conditions. Nevertheless, the total N contents were not different between the two cultivars at 14 d of treatment, regardless of N levels and tissues (Figure 4). It is well-known that, in many soils, particularly in those with annual crops such as wheat, NO_3_^−^ is the most abundant source of N and a preferred form for wheat root uptake. Moreover, NO_3_^−^ also serves as an important signal regulating plant growth by mediating the alteration of gene expression and metabolism pathway [29,30]. We therefore compared the NO_3_^−^ concentrations between two cultivars under both conditions. Again no difference at 14 d of treatment was observed between two cultivars under the same conditions in both roots and shoots (Figure 5). Considering all results, it is suggested that both total N content and NO_3_^−^ concentration may not be the major factors responsible for the higher chlorophyll content in Scout 66, while the signal derived from the NO_3_^−^ concentration changing could play a critical role in affecting chlorophyll metabolism.

Nitrogen absorption efficiency and N utilization efficiency are important indexes to evaluate the N use efficiency of plants, which were influenced by roots and shoots biomass, architecture, and responses to the deficiency nourishment environment [31,32]. Initially N, which is rapidly utilized, is taken up via seedling roots and is important for early crop establishment. Nitrogen continues to be taken up, driving the establishment of the canopy and the critical photosynthetic apparatus [31]. It has been reported that wheat classes differ on yield responses to N and, consequently, may be a factor responsible for the variation in absorption and utilization efficiency of N [33,34]. In our study, because both total N content and NO_3_^−^ concentration were not significant different under N deficiency between two cultivars; there was a significant difference in growth and biomass of root and shoot between both cultivars at different N levels. We subsequently compared both the absorption and utilization efficiency of N in roots between two cultivars (Figure 3), a common phenomenon that had been reported in various studies under N deficient responses [35,36]. The Similar responses were observed in our study regardless of cultivar (Figure 1). However, only a significant increase in root length was observed in Scout 66 under N-deficient conditions. Thus, we wondered whether the absorption and utilization of N by the two cultivars was different. Overall, the root N absorption efficiency gradually reduced, but the root N utilization efficiency gradually increased in both cultivars, particularly over long-term treatments (Figure 3). The elongation of N stress time and the fact that the roots absorbed and accumulated less N also lead to the increase of N utilization efficiency [37]. Nevertheless, this phenomenon differs in different genotype cultivars, as it was showed that Scout 66 had higher root N absorption efficiency in the roots while Atlas 66 showed higher N utilization efficiency in the roots in response to N deficiency (Figure 3). But over the treatment time points, there were no significant differences in N contents in roots except during the middle time points (Figure 4A), as well as that total N accumulation of Scout 66 in the roots was higher than that of Atlas 66 (Figure 4C). In this study, it was found that the increased root length of Scout 66 under low N stress might have resulted in the increased accumulation of N in the root system. For Atlas 66, the increased N utilization efficiency of roots may also be an important mechanism for its response to low N stress, but it might be not related to total N accumulation in the root.

While there are plenty of studies with the molecular mechanism of root N uptake in model plant of Arabidopsis and rice [38,39,40], available information on wheat root N acquisition has long been limited due to the unavailability of the large and complex wheat genome sequences. Recently, after the release of the genome of common wheat [23,24] and the fact that *NRT2* gene expression at the transcription level belongs to the HATS family, the critical role of NO_3_^−^ acquisition from soil under N-limitation conditions has been shown [20]. Since some of the *NRT2* transporters require a small partner protein named *NAR2.1* (or *NRT3.1*) for their function [21]. So, we first determined the expression pattern of *TaNAR2.1* in the two cultivars under N deficiency over treatment time. Results showed that the expression of *TaNAR2.1* was overall significantly induced by N deficiency in both cultivars which is consistent with the results obtained in tomato. In Reference [41], it was found that the expression of *TaNAR2.1* was induced significantly in high N-use efficiency tomato cultivar Regina Ostuni (RO) and low N-use cultivar UC82. The reduction of treatment compared with control is higher in UC82 than that in RO [41]. In our study, the extent of induction was greater in Atlas 66 than in Scout 66 under long-term N starvation (Figure 6A), suggesting that Atlas 66 might be N-use efficiency cultivars, which is actually verified by our findings (Figure 3). According to above results for physiological and molecular traits, it seems that Atlas 66 could be used as N-efficiency cultivar due to the fact that induces the *TaNAR2.1* expression, upon N deficiency. As for how the increased expression of *TaNAR2.1* triggers the high N-use efficiency awaits further verification. Expression of three NO_3_^−^ transporters (*TaNRT2.1, TaNRT2.3,* and *TaNRT2.4*) of the *TaNRT2* gene family was also analyzed, and the results showed that the expression of all the examined transporters were dramatically decreased in both cultivars over the N starvation period, except at 6 h of N stress (Figure 6B–D), which is against the expression patterns in tomato. It was shown that, upon N shortage for two weeks, the expression of both *TaNRT2.1* and *TaNRT2.3* was upregulated [41]. Moreover, *TaNRT2.1* was induced greater in UC82, while *TaNRT2.3* was induced more in RO. Interestingly, in our study, at the treatment time point of 6 h, expression of these genes was significantly induced in both cultivars and was decreased gradually from 1 d of N stress and onwards. This result suggest that the *TaNRT2* gene family could respond to N deficiency very fast and was induced in the early treatment, whereas those transporters might be late N responsive and need long-term N starvation to fully induce their expression. It was noticed that at the 14 d time point, the extent of expression of these transporters was more significant in Scout 66 than that of Atlas 66 when the N supply was sufficient (Figure 6B–D). In tomato, under N sufficiency, the steady-state transcript levels of these transporters were slightly increased but not significantly higher in RO [41]. While in our study, physiological determination showed that Scout 66 had higher root N absorption efficiency (Figure 3). It remains to be explored in the future whether the N-acquisition efficiency is either related to the basic transcript levels of NO_3_^−^ transporters or related to their induction magnitude or induction time.

A proteomic approach is a powerful high throughput technique to obtain a global review of the biological process and metabolic pathway by means of analyzing the abundance changed proteins under stresses. To take insight into the molecular mechanism of how different wheat cultivars respond to N deficiency, a label-free based quantitative proteomic study was conducted in the N sufficient and deficient wheat leaves in both cultivars. Overall, the total numbers of identified proteins were comparable (Figure 7) in two cultivars, suggesting the genotype difference does not substantially affect the global proteome composition, particularly under normal conditions. However, the numbers of the abundance changed proteins upon N deficiency in two cultivars were noticed, with as more as 76 proteins being affected by N deficiency in Scout 66 (Supplementary Materials, Table S1,S2). This is mainly because there are more than 53 downregulated proteins in Scout 66 upon N deficiency. It is suggested that the global proteome composition of Scout 66 might be more responsive to N deficiency, which could be a factor responsible for the higher chlorophyll content in Scout 66. Indeed, MapMan analysis uncovered that the metabolic pathway tetrapyrrole synthesis was the most enriched in Scout 66, while it was only relatively enriched in Atlas 66 (Table 1 and Figure 11A,B). In addition, it has been known that N and sulfur (S) co-exist in the biosphere either as free elements or as the simple inorganic NO_3_^−^ and SO_4_^2−^ oxyanions, both of them must be first reduced before undergoing anabolic processes, finally leading to the production of methionine (Met) and other S-containing molecules [42]. Studies have shown that N metabolism will feedback regulate S metabolism and both N and S pathways are tightly regulated in plant tissues so as to maintain S:N ratios ranging from 1:20 to 1:35 [42,43]. While increasing evidence has proved productions of S metabolism are associated with the adaptation of plants to various environmental stresses [44,45], indicating the critical role of S metabolism. Interestingly, in this study, MapMan analysis revealed that, upon N deficiency, sulfate assimilation was specially enriched in Scout 66, and the involved proteins were downregulated (Table 1 and Figure 11D). This result suggests that Scout 66 could be better in adjusting the S:N ratio and maintaining a relatively well cellular metabolism of various ions thus resulting in a better adaptation to stress. Of course, this hypothesis is still awaiting more studies in the future. Nevertheless, the interplay between S and N is worthy of further study, particularly in wheat, because the content of S-containing amino acids of wheat grain protein is also tightly correlated to flour quality and storage.

## 4. Materials and Methods

### 4.1. Plant Materials and Low Nitrogen Treatment

Healthy and disease-free seeds of two wheat cultivars (*Tritium aestivum* L.) Atlas 66 (Al-tolerant) and Scout 66 (Al-sensitive) were collected and surface-sterilized with 10% (*v*/*v*) H_2_O_2_ for about 30 min and rinsed thoroughly with deionized water five times, then soaked in deionized water for 2 h in room temperature. Next the soaked seeds were moved onto filter papers in a Petri dish for germination with deionized water in darkness at 26 °C for 3 days. The germinated seedlings were exposed to light, and similar seedlings were transferred into a plastic box (1 L) for about 2 weeks until the third leaves were fully developed in Modified Hoagland (MH) solution (1/4th strength) which was composed of: Ca (NO_3_)_2_∙4H_2_O (2 mM), MgSO_4_∙7H_2_O (650 μM), KH_2_PO_4_ (250μM), K_2_SO_4_ (750 μM), MnSO_4_∙H_2_O (10 μM), CuSO_4_∙5H_2_O(0.1 μM), ZnSO_4_∙7H_2_O (1 μM), H_3_BO_3_ (1 μM), (NH_4_)_6_MoO_24_∙4H_2_O (0.05 μM), KCl (100 μM), Fe-EDTA (40 μM). The pH of the solutions was adjusted to 6.0, replenishing regularly every 3 d. The N-sufficient nutrient solution (+N) and N-deficient nutrient solution (−N) contained Ca(NO_3_)_2_∙4H_2_O of 2 mM and 50 µM, respectively. All experiments were conducted in a controlled-environmental chamber (light/dark cycle of 14/10 h), the light intensity of 250 µmol photon m^−2^ s^−1^, the temperature at 26 °C, and 65% relative humidity.

### 4.2. Physiological Assays

The root and shoot samples were collected at 10 am for each scheduled time point including 6 h, 1 d, 3 d, 7 d and 14 d in both N levels. The root samples were rinsed several times with deionized water to remove the surface ions and blotted dry with tissue papers. The root and shoot lengths of the samples were first recorded and then oven dried at 72 °C for 60 h for measurement of sample dry weight.

### 4.3. Determination of Total Nitrogen and Nitrate Concentrations

The dried root and shoot samples were digested with H_2_SO_4_-H_2_O_2_, and the total N content was determined following the method described in Reference [46]. Fresh samples were immediately put into the liquid nitrogen to measure nitrate (NO_3_^−^) concentration [47]. Others parameters were calculated based on the following formula:

Total N accumulation = root dry weight × N content of root + shoot dry weight × N content of shoot
Root N absorption efficiency = total N accumulation/root dry weight
N utilization efficiency = plant dry weight/total N accumulation
Shoot N utilization efficiency = shoot dry weight/total N accumulation
Root N utilization efficiency = root dry weight/total N accumulation

### 4.4. Measurement of Chlorophyll Content

A weight of 0.2 g of fresh leaves was collected at 14 d time point and was put in plastic vials, followed by adding 10 mL of 80% (*v*/*v*) acetone into each vial. The plastic vials were placed at room temperature in the dark overnight; subsequently, the total chlorophyll contents were determined according to the method by Arnon [48]. The chlorophyll a and chlorophyll b contents were measured at the wavelengths of 663 nm and 646 nm, respectively. The chlorophyll content was expressed based on fresh weight (mg/g FW).

### 4.5. Protein Extraction and Determination of Protein Concentration

Total protein was extracted from leaf samples following the methods described by Lan et al. [49] with slight modifications. Briefly, each leaf sample with 0.2 to 0.04 g was fine ground into powder with liquid N and transferred into a 50 mL centrifuge tube (BECKMAN COULTER^®^), followed by addition of 20–40 mL pre-cooled acetone containing 10% (*v/v*) TCA and 0.07% (*v*/*v*) into each tube and vortexed thoroughly. Subsequently, the tubes were saved at −20 °C freezer at least 2 h to precipitate the total proteins. Later, 15 mg crude protein pellets from each sample were transferred into a 1.5 mL centrifuge tube and dissolved in 250 µL SDT buffer consisting of 2%(*w*/*v*) SDS, 0.1M Tris/HCl (pH 7.6), 0.1 M dithiothreitols, and 1 mM PMSF to extract the total proteins on ice for around 2–3 h. Meanwhile, the mixture was vortexed every 30 min in order to mix properly. The mixture was then centrifuged at 13,000× *g* for 10 min at 4 °C, and the supernatant was collected as the total proteins and the protein concentration was then determined by a fluorescent assay of the tryptophan method as described in Reference [50].

### 4.6. Protein Digestion and Peptide Purification

The filter-aided sample preparation (FASP) method, described in Reference [51], was applied to digest the total proteins. Briefly, around 50 µg protein from each sample was taken and added in to 10K MWCO. 0.5 mL PierceTM protein concentration (Thermo Fisher Scientific). Followed, the SDT buffer used to dissolve total proteins will replaced twice by ultrafiltration with UA buffer which consists of 8 M urea, 100 mM Tris-HCl (pH 8.5), with 300 µL each times. The ultrafiltration was performed by centrifuge at 10,000× *g* for 30 min. Subsequently, 100 µL iodoacetamide (50mM in UA buffer) was added into each sample, shaken at 600 rpm for about 1 min, then incubated in the dark for 30 min at room temperature, followed by centrifuge at 10,000× *g* for 15 min. Then, in order to wash the filtration column, 200 µL UA buffer was repetitively added followed by centrifuge for at least three times. This step was again washed two times by 300 µL NH_4_HCO_3_ (50 mM). Finally, 1 µg Trypsin in 100 µL NH_4_HCO_3_ (50 mM) solution together with 1 µL 100 mM CaCl_2_ was added into each filtration column mentioned above, vortexed around 1 min. Thereafter these columns were incubated around 16–18 h at 37 °C for digestion of the total proteins. After digestion, filtrated were collected as the digested peptide mixtures by centrifuging at 10,000× *g* for 5 min and washed two times with 50 µL NH_4_HCO_3_ solution and pooled together and finally added 10% TFA solution to a final concentration of 0.4%.

Followed, the peptide mixtures from each sample will be further purified using PierceTM C-18 Spin columns. These columns were first pretreated with 200 µL methanol and centrifuged at 1000× *g* for 30 s. Thereafter, wash the columns successively with 200 µL buffer B (0.2% TFA) two times, and buffer A (64% acetonitrile, 0.2% TFA) three times and centrifuged at 1000× *g* for 30 s accordingly. Around 200 µL buffer A solution was used to dissolve the peptide samples and then loaded into the column and centrifuged accordingly at 1000× *g* for 30 s. The filtrate was collected, re-added into the column and centrifuged at 1000× *g* for 30 s. These steps were performed two times. After that, a total of 200 µL buffer A was added into the column and centrifuged at 1000× *g* for 30 s in order to wash the samples. This step was repeated three times. In order to elute the peptides, a total of 180 µL buffer B was added and centrifuged at 1000× *g* for 30 s and this step was repeated one times. Finally, the collected effluent was dried in a centrifugal speed vacuum concentrator, and the powder was kept at −80 °C.

### 4.7. LC-MS/MS Analysis

Desalted peptides dissolved in 20 µL (0.1%) formic acid were scanned in an Orbitrap Fusion Lumos mass spectrometer (Thermo Fisher Scientific) for NanoLC-ESI-MS/MS analysis. First, the dissolved peptide samples were carefully transferred to sample bottles and were sonicated to remove any possible air bubbles and then the sample bottles were placed on sample racks which were held in the carousel in a autosampler of the DIONEX UltiMate 3000 RSLCnano System (Thermo Fisher Scientific). Buffer A (0.1% formic acid in water) was used to balance the C18 analysis column (Acclaim PepMap^TM^ 100, 100 µm × 2 cm, Thermo Fisher Scientific) till the system is stable. Thereafter the peptides were automatically loaded onto the column and eluted with a flow rate of 0.3 µL/min with a linear gradient. The liner gradient was 85 min in total, starting from 0% to 35% buffer B (0.1% formic acid in acetonitrile) over a total of 70 min. Subsequently, buffer B was increased to 80% for about 1 min and continually maintained the same conditions for another 5 min. Mass spectra was used in full scan with an acquired range of 350–1700 m/z with a mass resolution of 60,000. In each MS scan, the 20 most intense peaks were selected and further fragmented with a higher-energy collisional dissociation with collision energy of 30% and the resolution of MS/MS spectra scan was set at 15,000. The mass spectrometer was run under positive mode. Three biological repeats were carried out for MS/MS analysis with two technical repeats for each biological repeat.

### 4.8. Database Search and Protein Quantification

The Proteome Discoverer software (version 2.3, Thermo Fisher Scientific) was used to identify and analyze differentially accumulated proteins (DAPs). After importing MS raw data and setting parameters (as shown below), searches were run against the IWGSC *Triticum aestivum* protein database (v1.1), downloaded from EnsemblPlants database. The searching parameters were set as follows: carbamidomethylation at Cys as a fixed modification, oxidation of methionine as variable modifications, trypsin as the digestion enzyme with two missed cleavages allowed, and a tolerance of 10 ppm for precursor and 0.02 Da for fragment ions. All datasets were analyzed using the workflow feature integrated in the Proteome Discoverer software.

For protein identification, the filters were set as below: the peptide confidence greater than 95% and Master protein confidence greater than 99%. These filters resulted in a protein FDR (false discovery rate) less than 1%, marked as High with Exp. q-value less than 0.01. To mine DAPs, only the confidential proteins, which must be found in at least one treatment as High after filter, were used and analysis was accomplished by means of label-free quantitative methods integrated in Proteome Discoverer software. A protein with fold change of ≥ 2 and adjusted *p*-value < 0.05 was defined as a DAP.

### 4.9. Bioinformatics Analyses and Visualization

The web tool agriGO was used to analyze Gene Ontology (GO) annotation and enrichment analysis with filtering threshold with adjusted *p*-values less than 0.05 and an intersectional set size of between 10–500 [52]. Biological pathway (BP) enrichment results were visualized by the EnrichementMap [53] and AutoAnnotate applications in Cytoscape as described in Reference [54]. Metabolic pathways were analyzed by MapMan version 3.1.1 [55].

### 4.10. Expression Analysis of Different Nitrate^−^Transporters by Quantitative Real-Time PCR (qRT-PCR)

The quantitative real time-PCR analysis was performed to analyze the transcriptional levels of genes. To perform *qRT-PCR*, samples were collected in the scheduled time points and immediately kept in liquid nitrogen and subsequently stored at −80 °C for further analysis. Total RNAs were extracted with TRIzol reagent (Invitrogen, Karlsruhe, Germany) according to the manufacturer’s instructions. The quantity of the RNAs was determined with NanoDrop (Thermo Fisher Scientific, NanoDrop 2000 Spectrophotometer) and quality was examined by agarose gel electrophoresis. A total of 1 µg of RNA was used to convert into cDNAs using Prime Script reverse transcriptase (Invitrogen, Karlsruhe, Germany). The SYBR green PCR Master Mix (Takara) was used to identify mRNA levels. The *qRT-PCR* program was set as follows: Pre-denaturation at 95 °C for the 30s, 40 cycles of denaturation at 95 °C for 5s and annealing at 60 °C for 30 s, followed by melt-curve analysis (60 °C, 95 °C, 0.5 °C increments for 5s). The relative expression levels of the studied genes were calculated according to the 2−ΔCT method [56]. The primers used in this study were listed in Supplementary Materials Table S8. The wheat *TaAPT1* (Adenine phosphoribosyltransferase 1) gene was used as a housekeeping gene.

### 4.11. Statistical Analysis

All the recorded values are the means of the results of three technical repeats for each treatment, and each parameter has at least three biological repeats. The SPSS statistical software (SAS Institute, Cary, NC, USA), version 22, was used to perform analysis of variance using a two-way analysis of variance (ANOVA). Significant differences among the treatment values were identified by using the least significant difference (LSD) multiple range tests (*p* < 0.05).

## 5. Conclusions

In this study, two contrasting wheat genotype cultivars were chosen to explore their responses to N deficiency, with a series of morphological, physiological, and molecular characteristics being examined under N deficient conditions. The results showed that different Al-sensitive cultivars exhibited different responses under N deficiency in terms of root length, chlorophyll content, and the expression patterns of N-responsive NO_3_^−^ transporters. Overall, the proteome compositions of the two cultivars were comparable, but a high number of changed proteins in Scout 66 were identified than that in the cultivar Atlas 66. Although most of the changed proteins were involved in or associated with the same biological processes or metabolic pathways, S assimilation was particularly enriched in Scout 66, emphasizing the importance of S:N balance under N deficiency and setting the following experimental stage to uncover the underlying mechanisms of why different Al-responsive cultivars show distinct responses to N deficiency and probably S status as well.

## Figures and Tables

**Table 1:** Differential accumulated proteins (DAPs) of Atlas 66 and Scout 66 enriched in tetrapyrrole synthesis, N metabolism, secondary metabolism flavonoids, and sulfate assimilation.

Cultivar	Accession	Function	Abundance Ratio: (−N) 14 d/(+N) 14 d	Abundance Ratio Adj. *p*-value: (−N) 14 d/(+N) 14d	Abundance Ratio Variability (%): (−N) 14 d/(+N) 14 d	Metabolic Pathway
Atlas 66	TraesCS7B02G314800.1	ALA dehydratase	100	9.00943820224719E-17	0	Tetrapyrrole synthesis
	TraesCS6D02G132500.2	Porphobilinogen deaminase	0.434	0.027271111	0.11	
	TraesCS2D02G563600.1	Protochlorophyllide reductase	0.01	9.00943820224719E-17	0	
	TraesCS2A02G590600.1	Protochlorophyllide reductase	0.153	2.55508858168777E-07	0.4	
	TraesCS1A02G171000.1	Protochlorophyllide reductase	0.403	0.0352935604237759	2.4	
	TraesCS4D02G081500.1.cds1	Regulation	0.448	0.0329941173590985	0.56	
Scout 66	TraesCS7B02G314800.1	ALA dehydratase	100	8.5793991416309E-17	0	
	TraesCS1B02G075200.1	Glu-tRNA synthetase	0.01	8.5793991416309E-17	0	
	TraesCS6A02G102500.1	Glu-tRNA synthetase	0.01	8.5793991416309E-17	0	
	TraesCS7D02G261800.1	GSA(Glutamate-1-semialdehyde aminotransferase)	0.01	8.5793991416309E-17	0	
	TraesCS7D02G062900.1.cds1	Magnesium protoporphyrin IX methyltransferase	0.01	8.5793991416309E-17	0	
	TraesCS2D02G563600.1	Protochlorophyllide reductase	0.01	8.5793991416309E-17	0	
	TraesCS2A02G590600.1	Protochlorophyllide reductase	0.15	3.40978161045871E-09	0.32	
	TraesCS1A02G171000.1	Protochlorophyllide reductase	0.372	0.00420395928177109	3.03	
	TraesCS1D02G168700.2	Protochlorophyllide reductase	0.401	0.00463374616837516	7.79	
	TraesCS5D02G364100.1	Unspecified	100	8.5793991416309E-17	0	
	TraesCS3D02G228800.1	Uroporphyrinogen decarboxylase	0.498	0.0491864992180654	4.99	
Atlas 66	TraesCS6A02G017500.2	Nitrate reductase	0.01	9.00944E-17	0	N metabolism
	TraesCS2D02G388800.1	Glutamate dehydrogenase	3.763	0.023094541	9.7	
	TraesCS5B02G437100.2	Glutamate dehydrogenase	100	9.00944E-17	0	
	TraesCS6B02G327500.1	Glutamine synthetase	4.162	0.01132758	12.21	
	TraesCS6A02G333900.1	Nitrite reductase	0.095	4.82064E-10	0.17	
	TraesCS6B02G364600.1	Nitrite reductas	0.219	9.96743E-05	0.5	
	TraesCS6D02G313100.1	Nitrite reductase	0.274	0.000867051	0.28	
Scout 66	TraesCS6A02G017500.2	Nitrite reductase	0.01	8.5794E-17	0	
	TraesCS5B02G437100.2	Glutamate dehydrogenase	100	8.5794E-17	0	
	TraesCS2D02G388800.1	Glutamate dehydrogenase	3.418	0.016723292	1.57	
	TraesCS6B02G327500.1	Glutamate dehydrogenase	3.47	0.004060041	17.17	
	TraesCS5B02G414000.3	Nitrate transporter	100	8.5794E-17	0	
	TraesCS6B02G364600.1	Nitrite reductase	0.298	0.000655234	2.21	
	TraesCS6A02G333900.1	Nitrite reductase	0.183	1.33016E-06	0.07	
	TraesCS6D02G313100.1	Nitrite reductase	0.324	0.000403989	1.26	
Atlas 66	TraesCS4A02G437900.1	Naringenin-chalcone synthase	11.196	3.95457E-05	48.59	Secondary metabolism flavonoids
	TraesCS1A02G021100.1.cds1	Anthocyanin 5-aromatic acyltransferase	100	9.00944E-17	0	
	TraesCS1D02G339200.1.cds1	Dihydroflavonols	100	9.00944E-17	0	
	TraesCS4B02G335300.2	Flavonols	100	9.00944E-17	0	
	TraesCS5D02G476400.1.cds1	Dihydroflavonols	100	9.00944E-17	0	
	TraesCS3B02G013100.1.cds1	Dihydroflavonols	100	9.00944E-17	0	
	TraesCS4A02G436200.1.cds1	Naringenin-chalcone synthase	100	9.00944E-17	0	
	TraesCS3D02G001400.1.cds1	Dihydroflavonols	100	9.00944E-17	0	
Scout 66	TraesCS1D02G324200.1	Sulfite reductase (SIR)	0.01	8.5794E-17	0	Sulfate assimilation
	TraesCS5B02G018700.2	OAS-TL A(O-acetylserine sulfhydrylase, O-acetylserine (Thiol)-lyase) (CSase A))	0.324	0.000478246	0.56	
	TraesCS7D02G096700.1	OAS-TL A(O-acetylserine sulfhydrylase, O-acetylserine (Thiol)-lyase) (CSase A))	0.317	0.000903302	2.09	
	TraesCS2A02G184200.1	Adenylylsulphate kinase	0.01	8.5794E-17	0	
	TraesCS5D02G027600.1	OAS-TL A(O-acetylserine sulfhydrylase, O-acetylserine (Thiol)-lyase) (CSase A))	0.408	0.008948742	2.79	

## Supplementary Materials

Supplementary materials can be found at https://www.mdpi.com/1422-0067/21/6/2119/s1.
