# Supplementary material for: Physiological and Proteomic Dissection of the Responses of Two Contrasting Wheat Genotypes to Nitrogen Deficiency
Source: Int J Mol Sci. 2020 Mar 19;21(6):2119. doi: 10.3390/ijms21062119 (PMC7139514; doi:10.3390/ijms21062119)
Supplement: Supplementary file 1 [file ijms-21-02119-s001.zip › 新建文件夹 (2)/Supplementary Figure_word form-revised (round 2).docx]

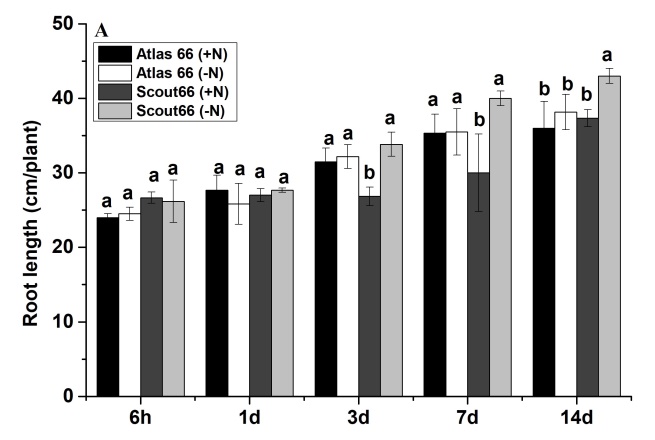

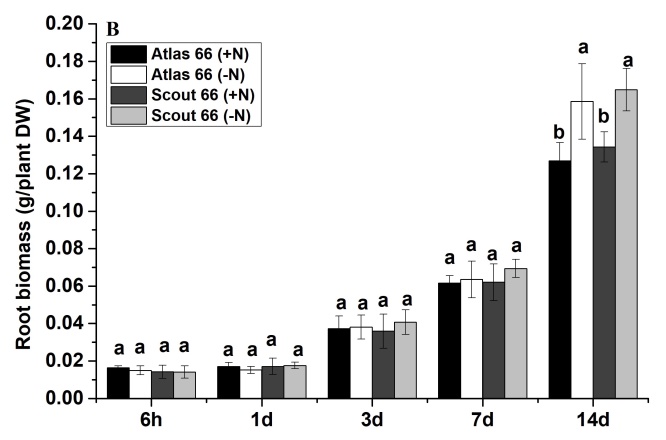


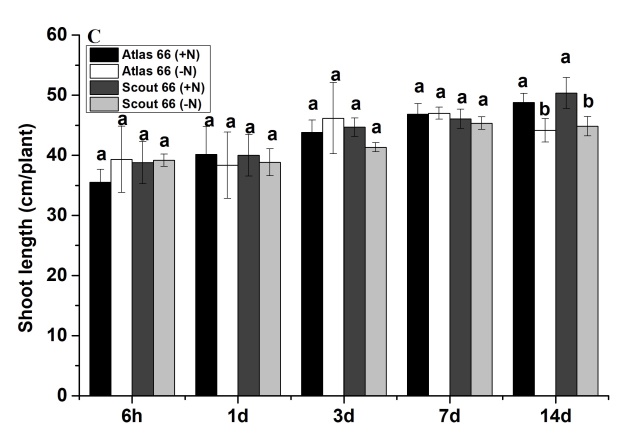

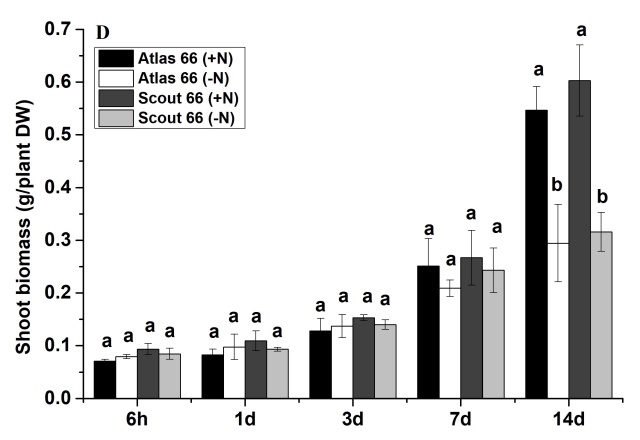


Supplementary Figure S1. Effect of N deficiency on wheat plant root length (A), root biomass (B), shoot length (C), and shoot biomass (D). Wheat plants were transferred from N sufficiency to either N sufficient (2 mM) or deficient (50 µM) conditions for defined treatment time and data presented here were sampled from 6 h to 14 d from both N levels. Bars represent the mean ±SD (*n*=3). Different small letters indicate statistically significant differences based on LSD test (*p* ≤ 0.05) among the treatments and cultivars.


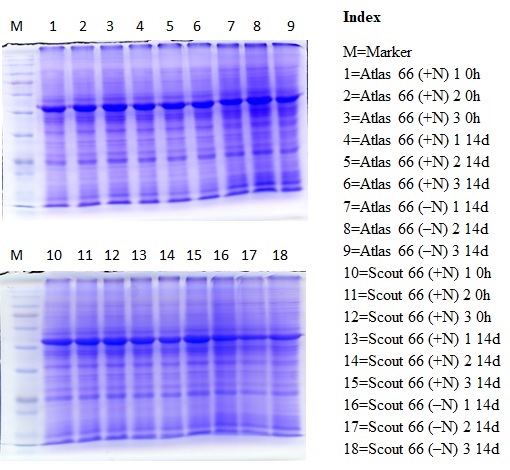


Supplementary Figure S2. Visualization of SDS-PAGE for quantification of proteins. In total 30 µg protein was used for running the SDS-PAGE and Coomassie Brilliant Blue was used to stain the protein band.


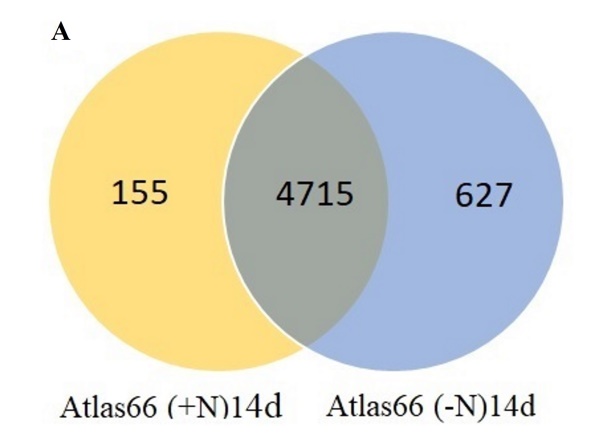

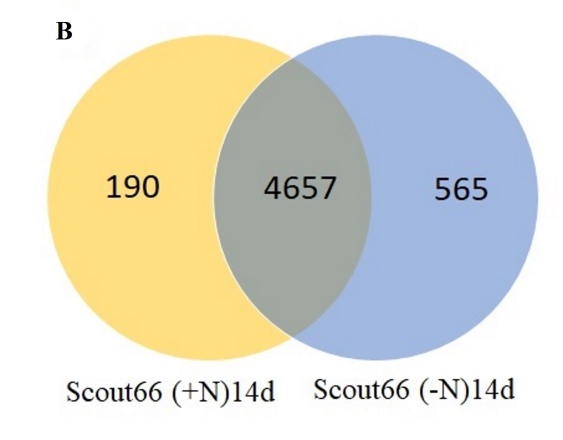


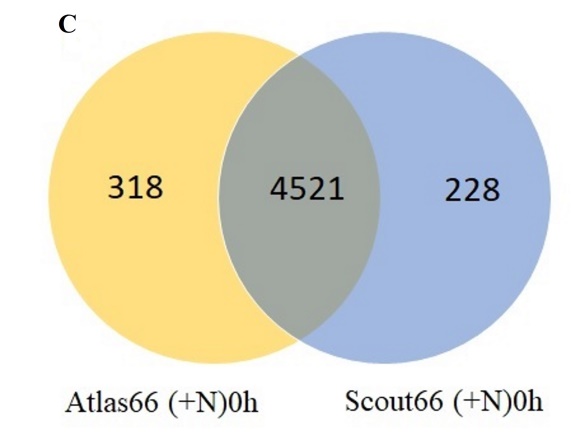

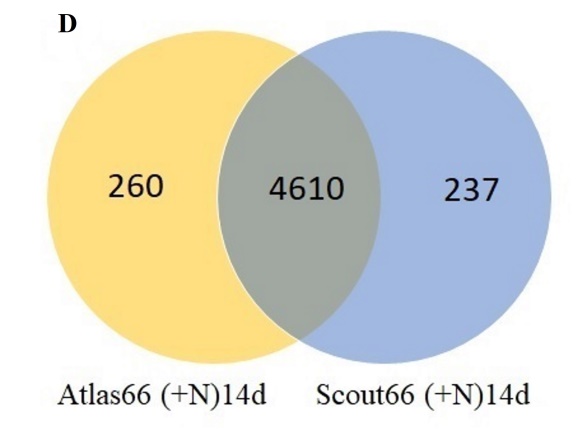


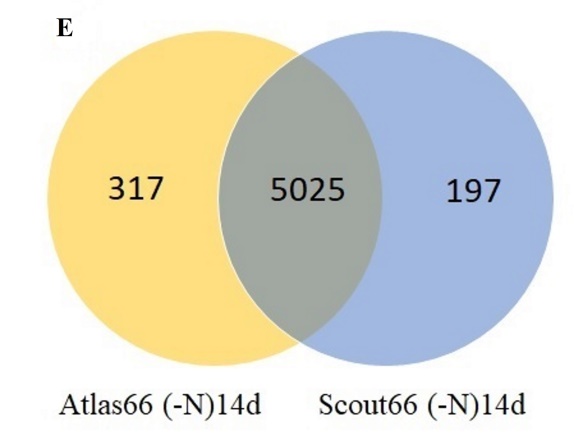


Supplementary Figure S3. Venn diagram of total proteins identified in leaves of Atlas 66 and Scout 66 in this study. The venn diagram showed the total proteins identified between two conditions Atlas 66 at (+N) 14d and Atlas 66 (-N) 14d (A), Scout 66 at (+N) 14d and Scout 66 (-N) 14d (B), Atlas 66 (+N) 0h and Scout 66 (+N) 0h (C), Atlas 66 (+N) 14d and Scout 66 (+N) 14d (D) , Atlas 66 (-N) 14d and Scout 66 (-N) 14d (E).


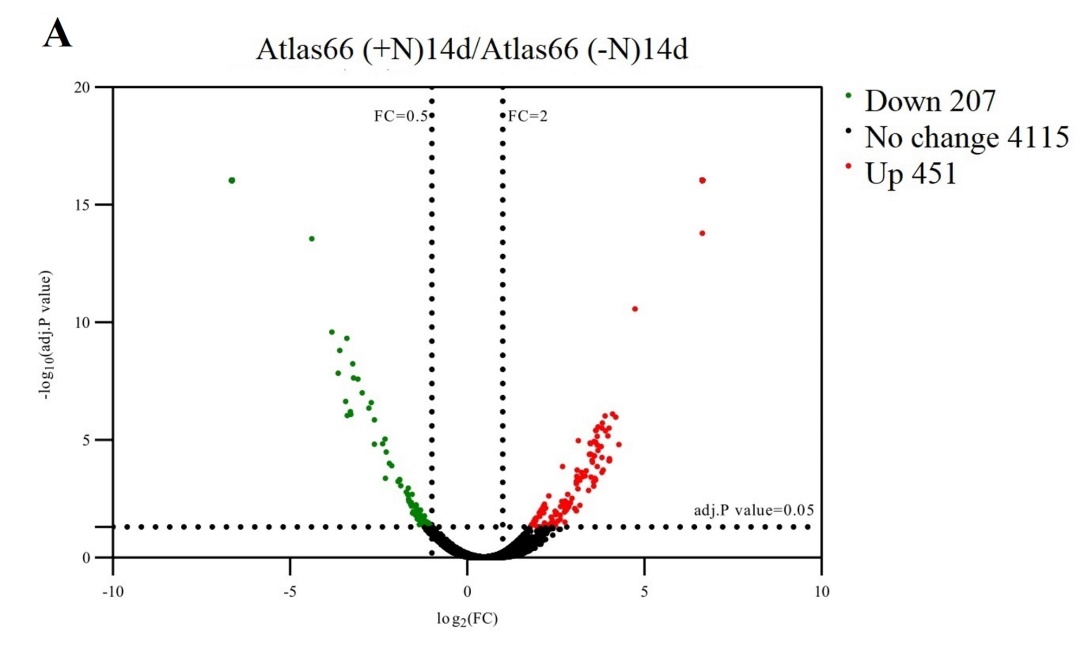


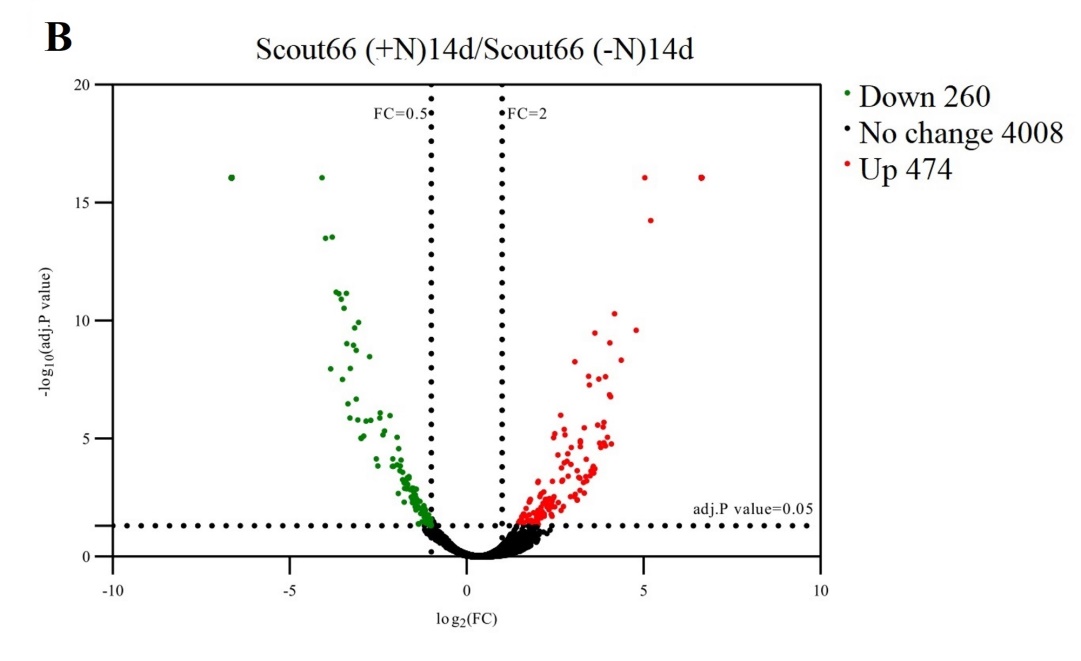


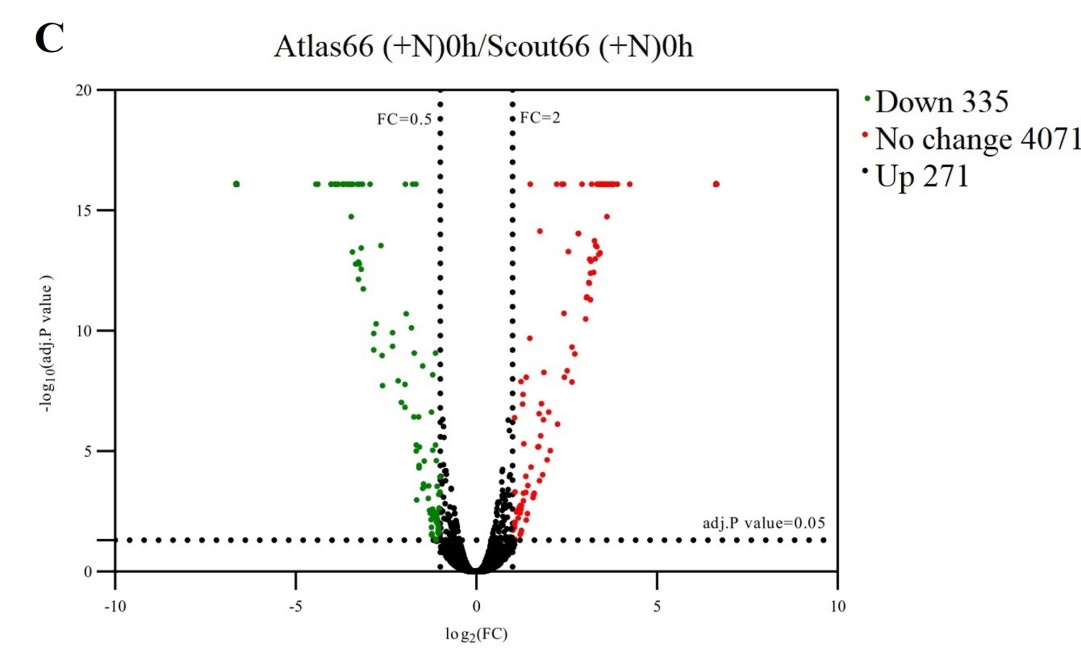


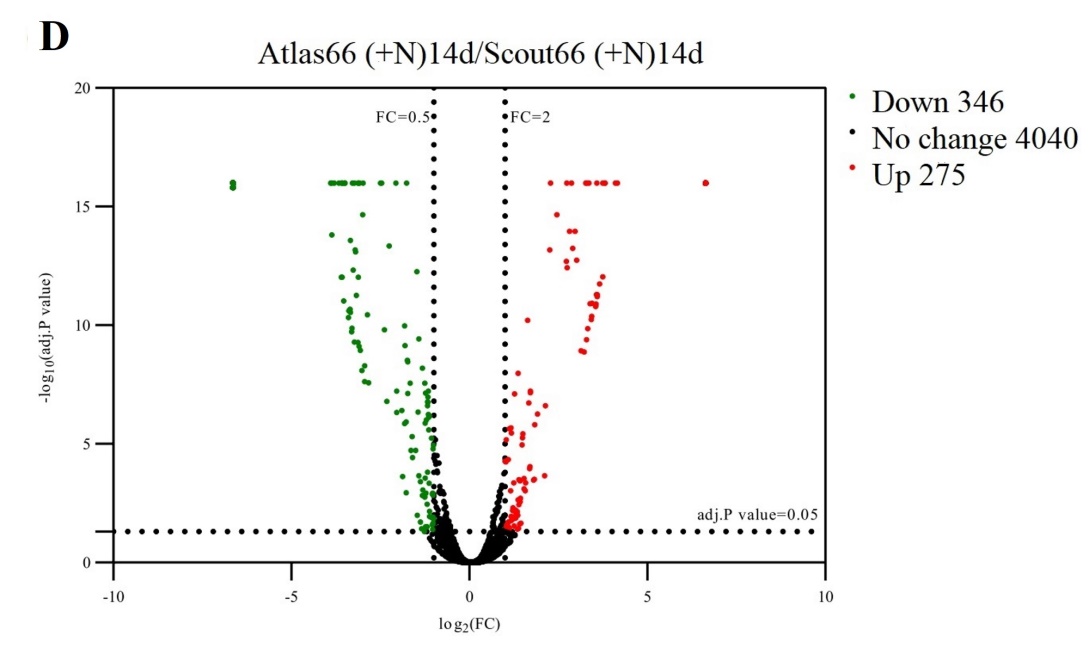

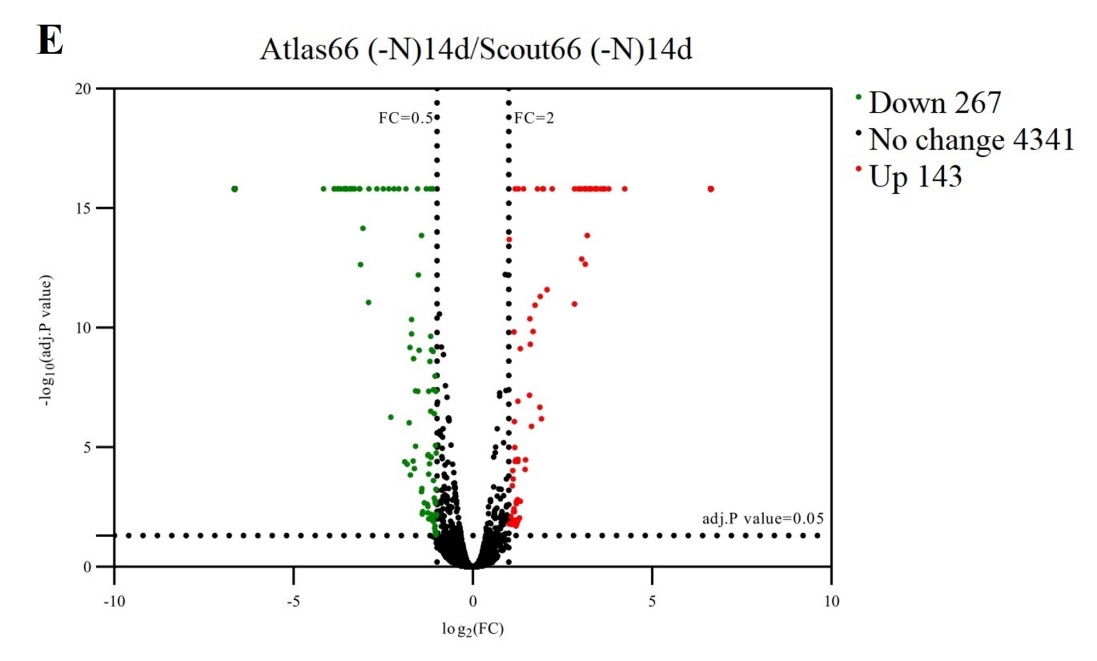


Supplementary figure S4 . Volcano plot of total proteins identified in leaves of Atlas 66 and Scout 66 in this study. The volcano plot showed the total proteins identified between two conditions Atlas 66 at (+N) 14d and Atlas 66 (-N) 14d (A), Scout 66 at (+N) 14d and Scout 66 (-N) 14d (B), Atlas 66 (+N) 0h and Scout 66 (+N) 0h (C), Atlas 66 (+N) 14d and Scout 66 (+N) 14d (D) , Atlas 66 (-N) 14d and Scout 66 (-N) 14d (E). The log_2_(FC) indicate a significant difference away from the zero (0) point. The –log_10_ (adj. P-value) represents adjusted P-value (*p* ≤ 0.05) in between the treatment combination


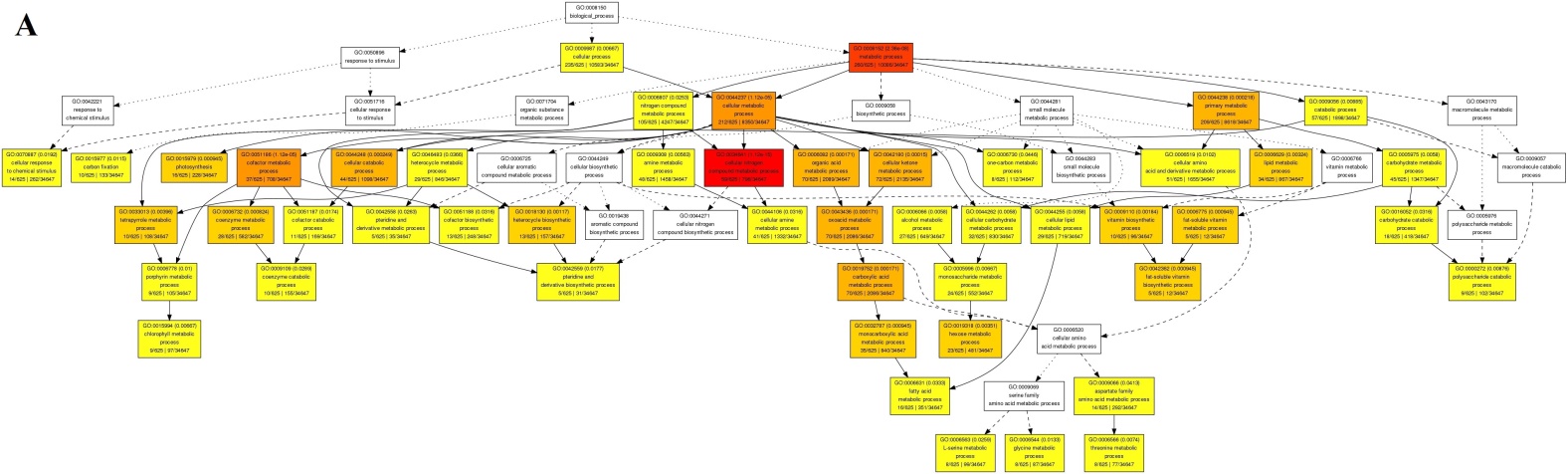


Supplementary Figure S5A. Gene Ontology (GO) analysis of DAPs in biological process in Atlas 66 between N deficiency and sufficiency


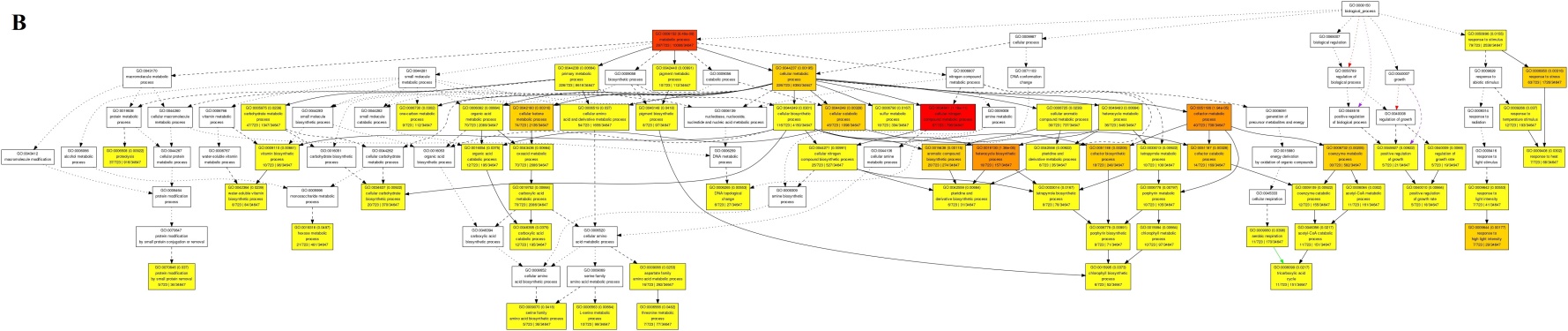


Supplementary Figure S5B. Gene Ontology (GO) analysis of DAPs in biological process in Scout 66 between N deficiency and sufficiency


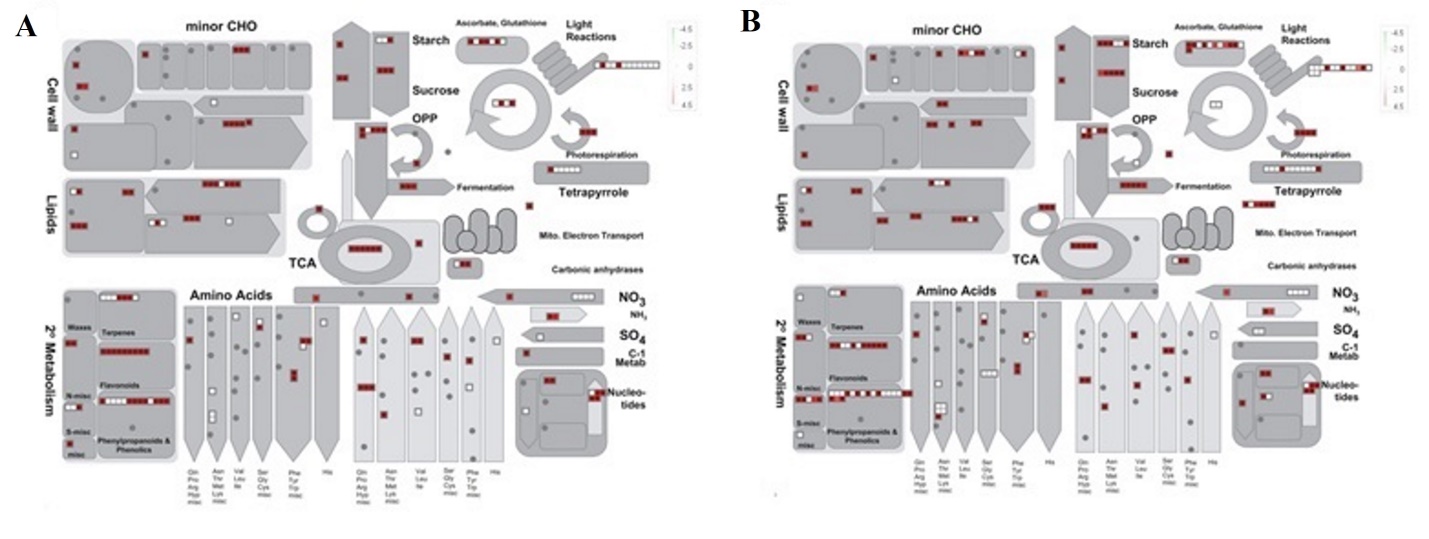


Supplementary Figure S6. Metabolism overview of the DAPs in Atlas 66 (A) and Scout 66 (B), they are analyzed in and exported from MapMan software.
