# Supplementary figures and images for: Physiological and Proteomic Dissection of the Responses of Two Contrasting Wheat Genotypes to Nitrogen Deficiency
Source: Int J Mol Sci. 2020 Mar 19;21(6):2119. doi: 10.3390/ijms21062119 (PMC7139514; doi:10.3390/ijms21062119)

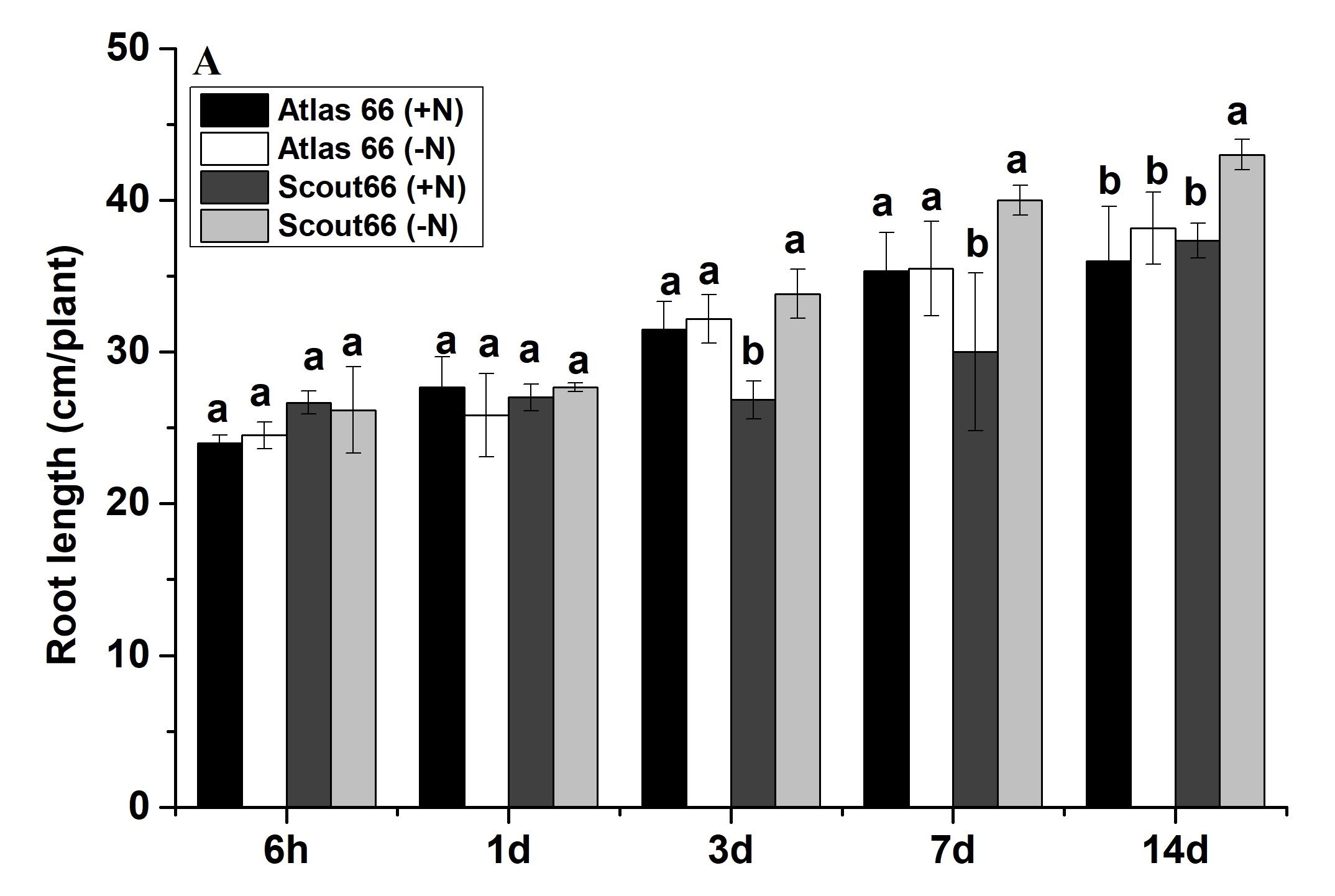

Supplement: Supplementary file 1 [file ijms-21-02119-s001.zip › 新建文件夹 (2)/Supplementary Figure_revised (round 2)/Supplementary Figure S1A.jpg]

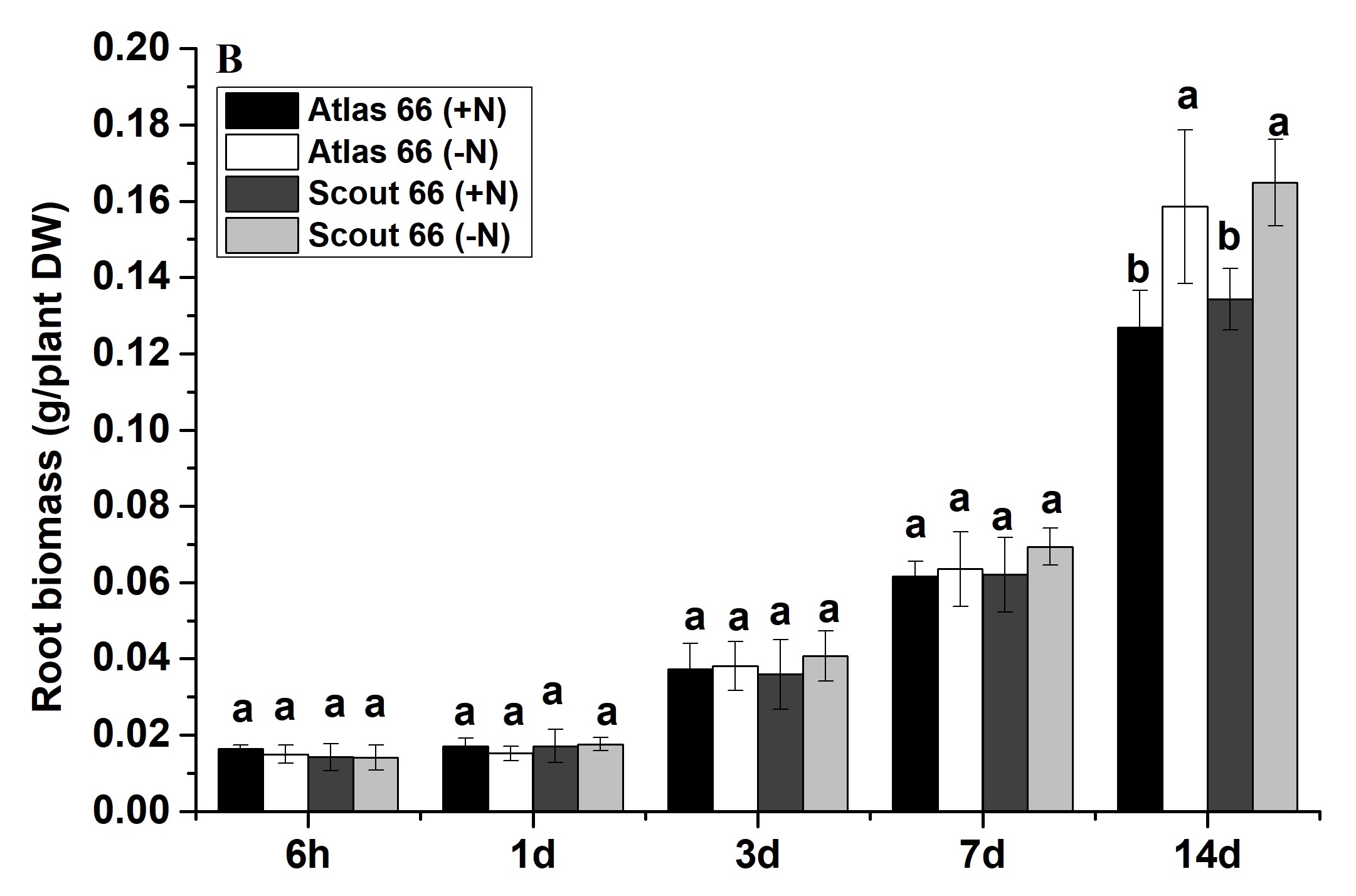

Supplement: Supplementary file 1 [file ijms-21-02119-s001.zip › 新建文件夹 (2)/Supplementary Figure_revised (round 2)/Supplementary Figure S1B.jpg]

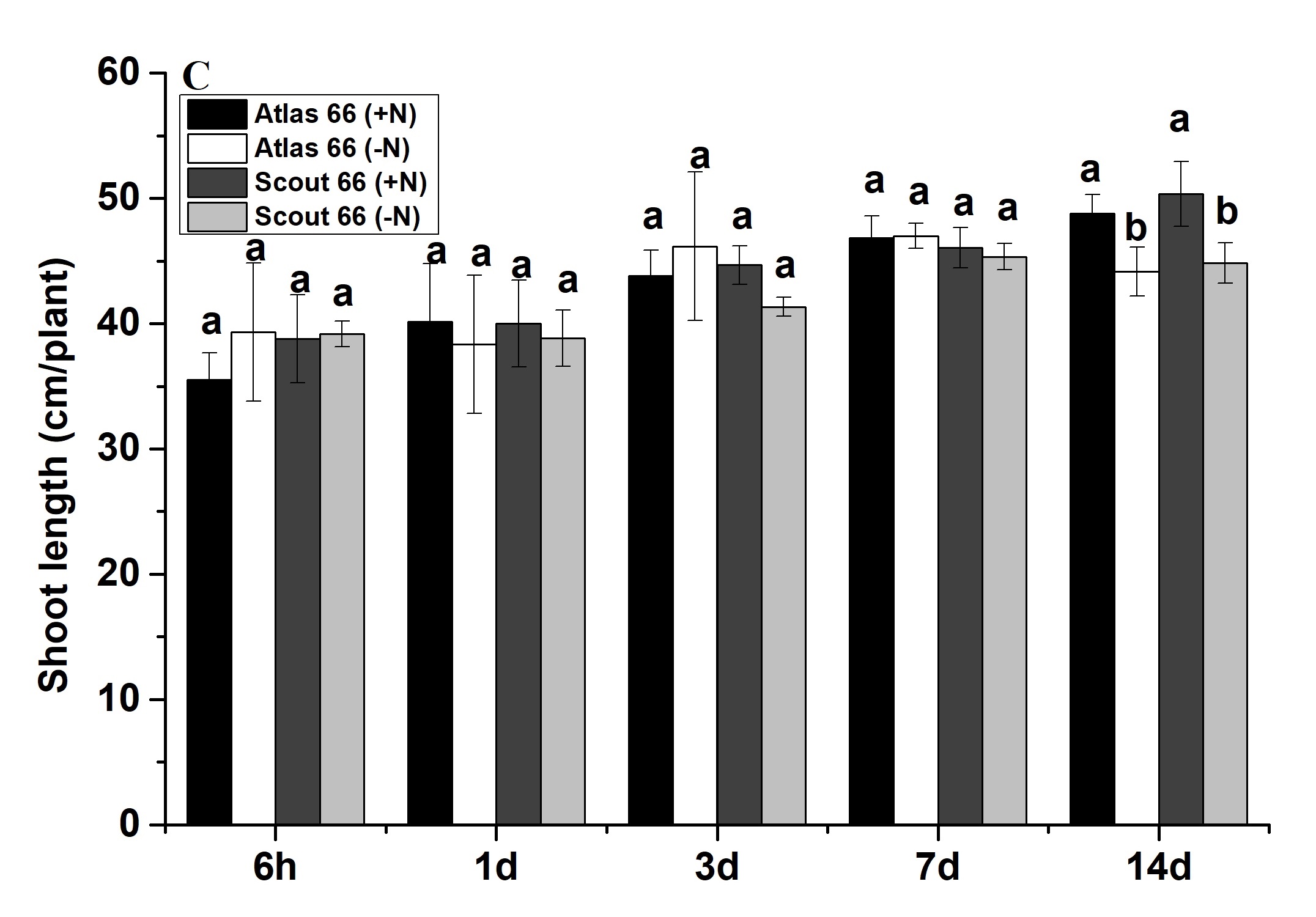

Supplement: Supplementary file 1 [file ijms-21-02119-s001.zip › 新建文件夹 (2)/Supplementary Figure_revised (round 2)/Supplementary Figure S1C.jpg]

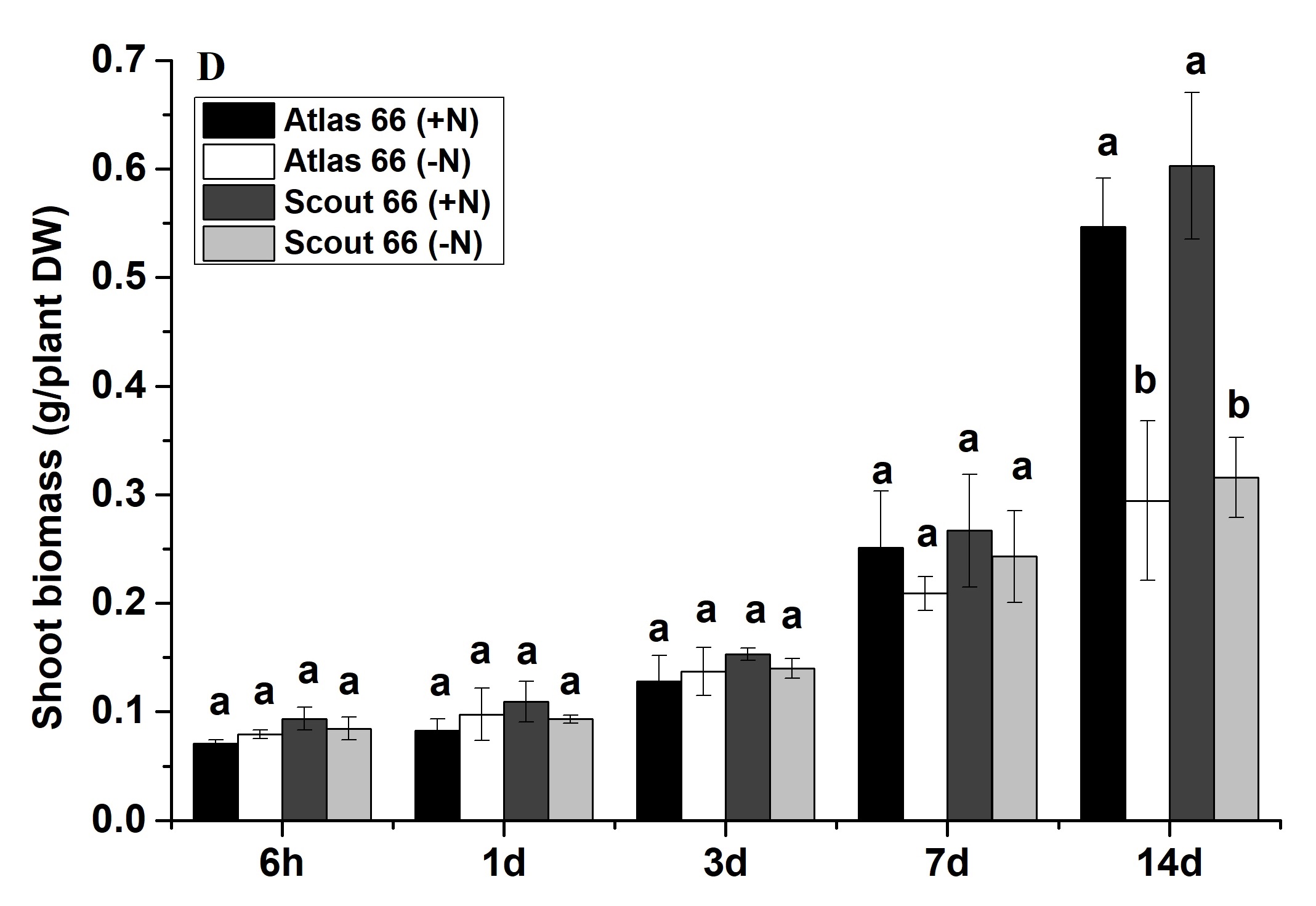

Supplement: Supplementary file 1 [file ijms-21-02119-s001.zip › 新建文件夹 (2)/Supplementary Figure_revised (round 2)/Supplementary Figure S1D.jpg]

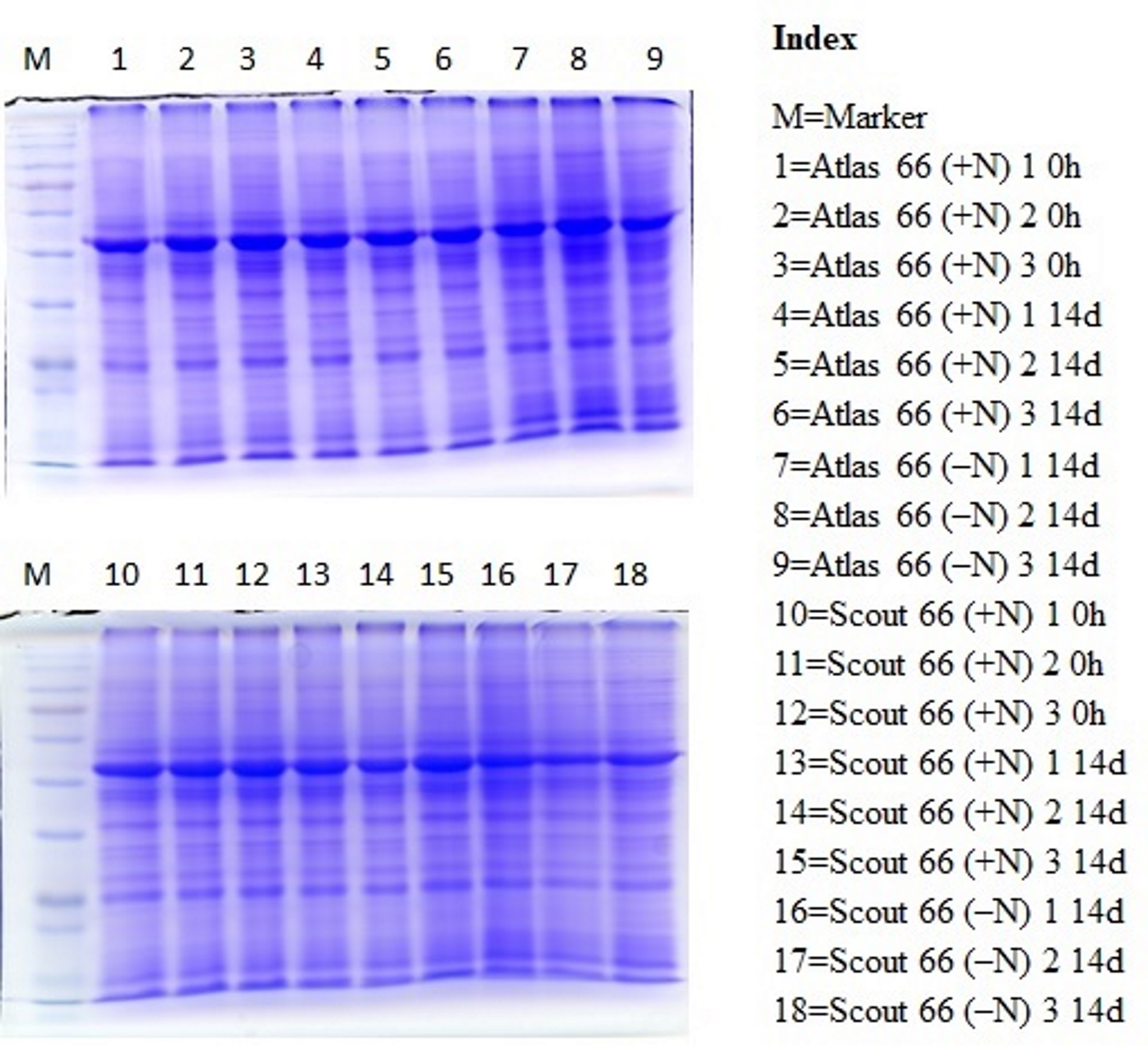

Supplement: Supplementary file 1 [file ijms-21-02119-s001.zip › 新建文件夹 (2)/Supplementary Figure_revised (round 2)/Supplementary Figure S2.jpg]

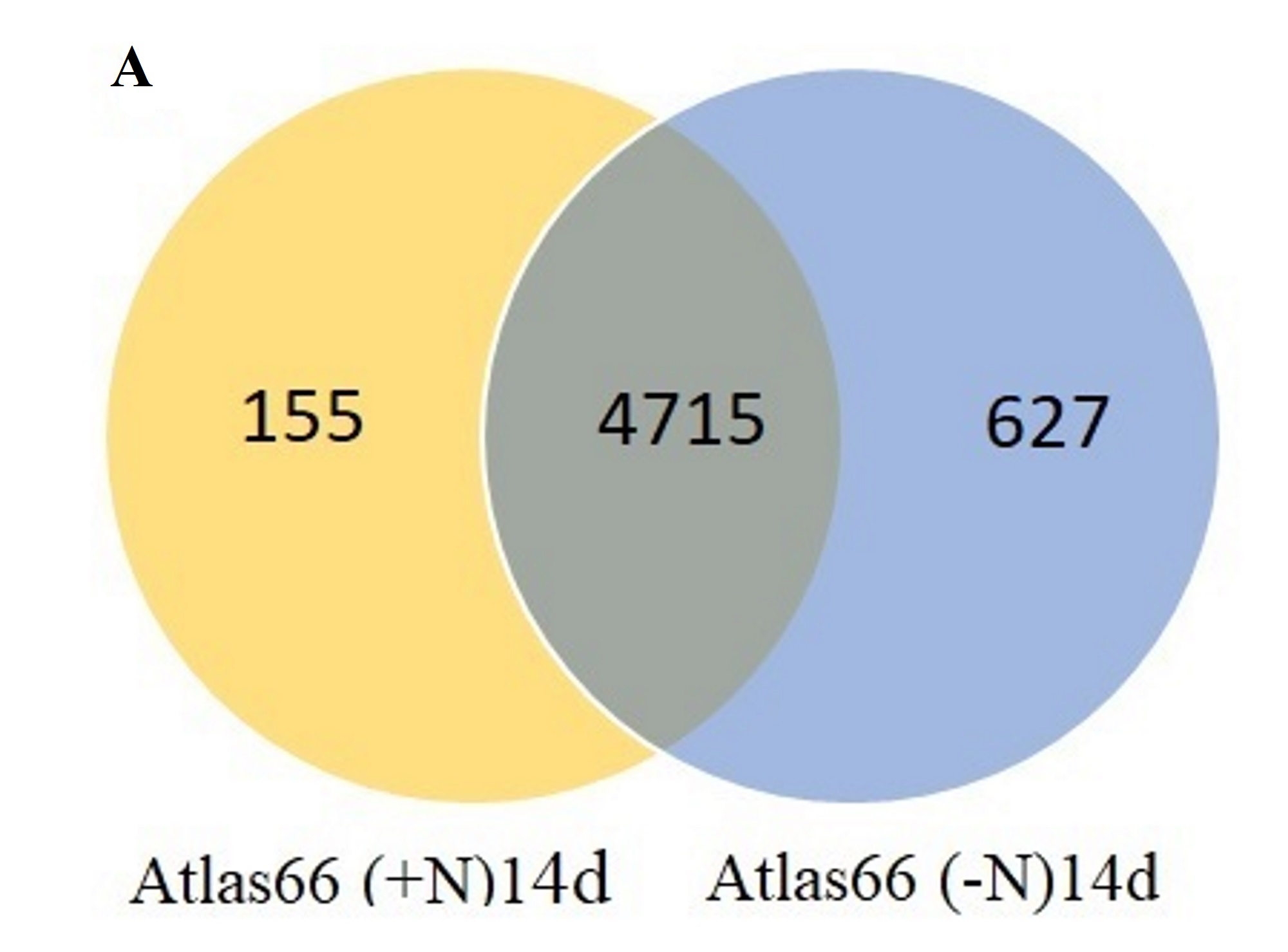

Supplement: Supplementary file 1 [file ijms-21-02119-s001.zip › 新建文件夹 (2)/Supplementary Figure_revised (round 2)/Supplementary Figure S3A.jpg]

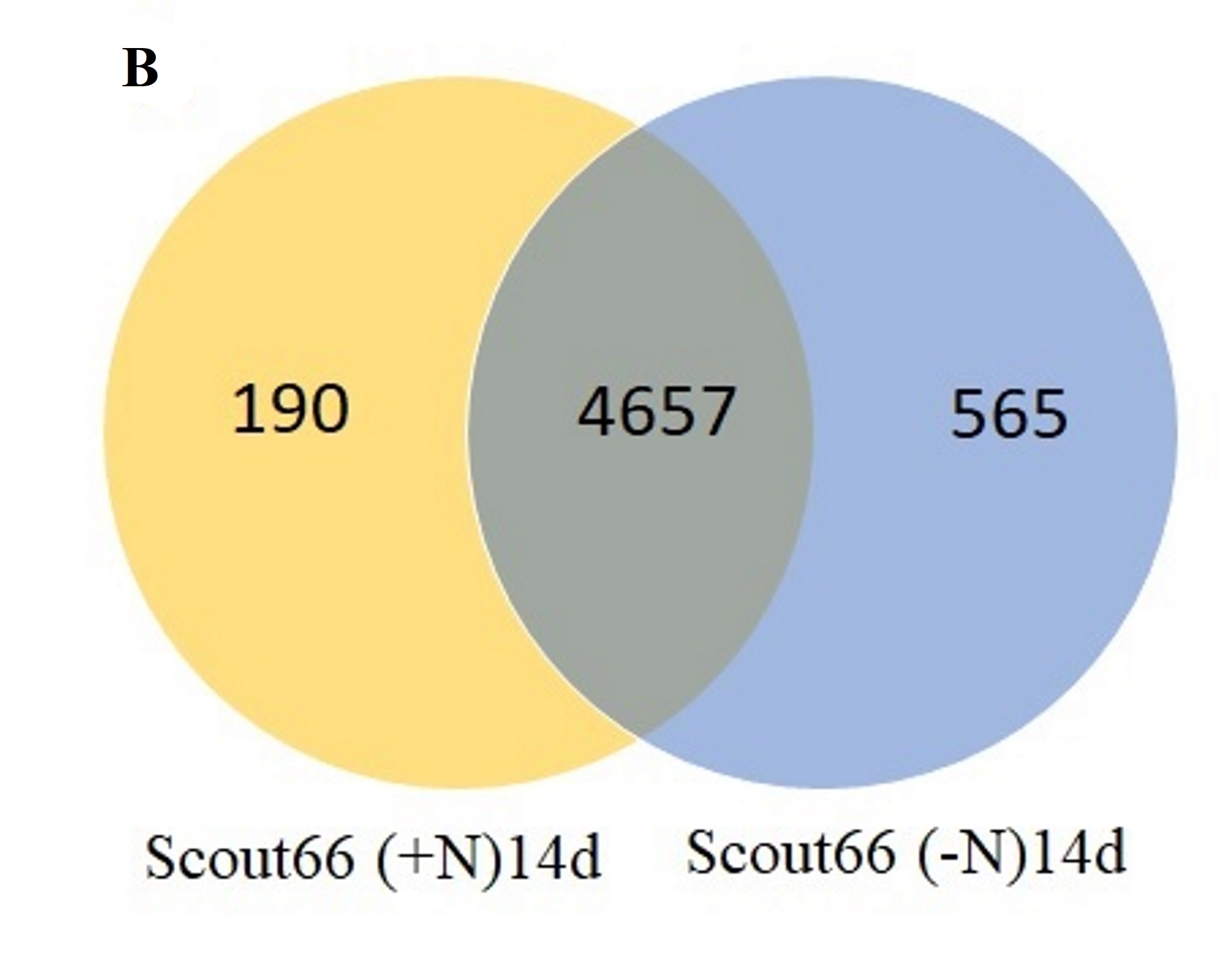

Supplement: Supplementary file 1 [file ijms-21-02119-s001.zip › 新建文件夹 (2)/Supplementary Figure_revised (round 2)/Supplementary Figure S3B.jpg]

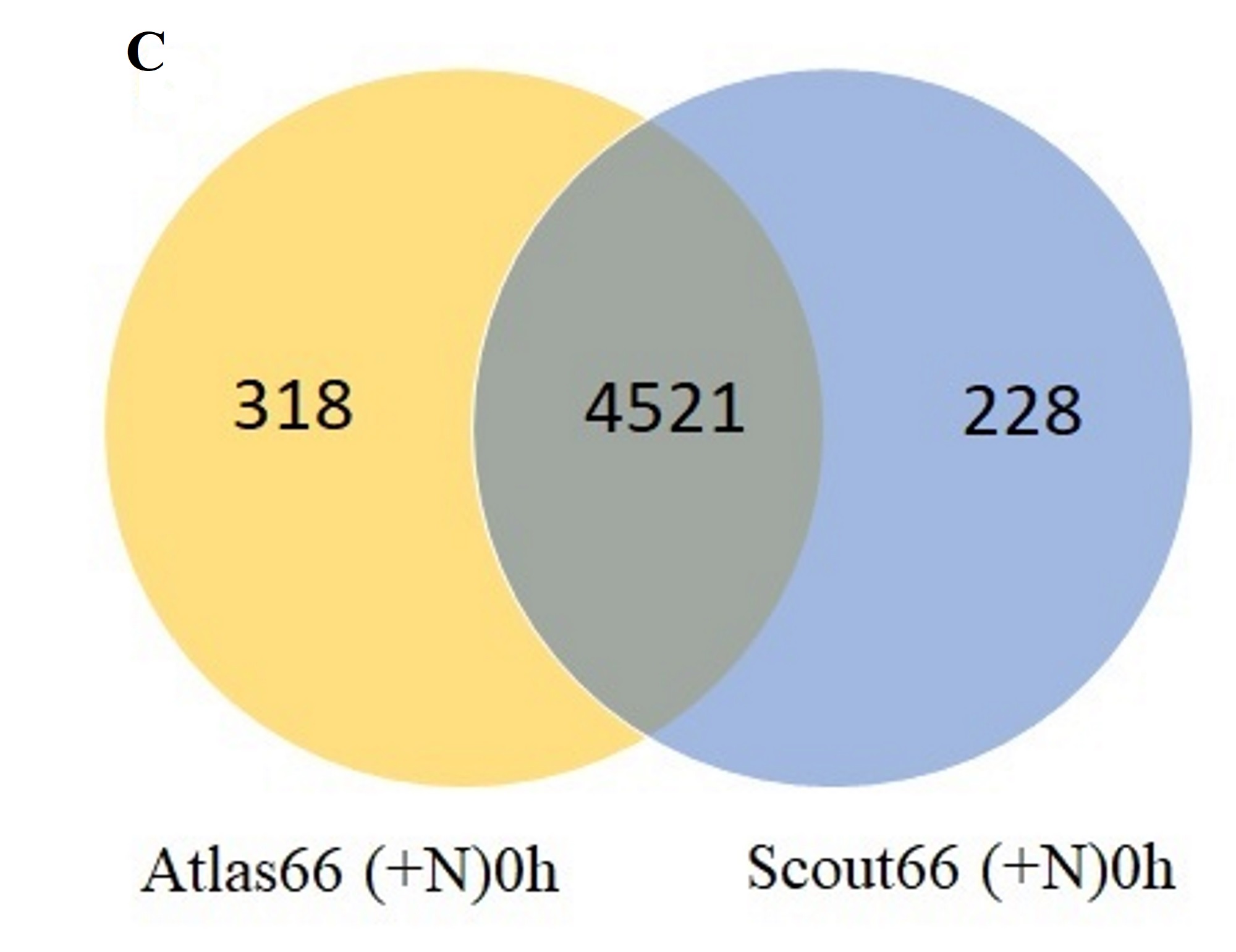

Supplement: Supplementary file 1 [file ijms-21-02119-s001.zip › 新建文件夹 (2)/Supplementary Figure_revised (round 2)/Supplementary Figure S3C.jpg]

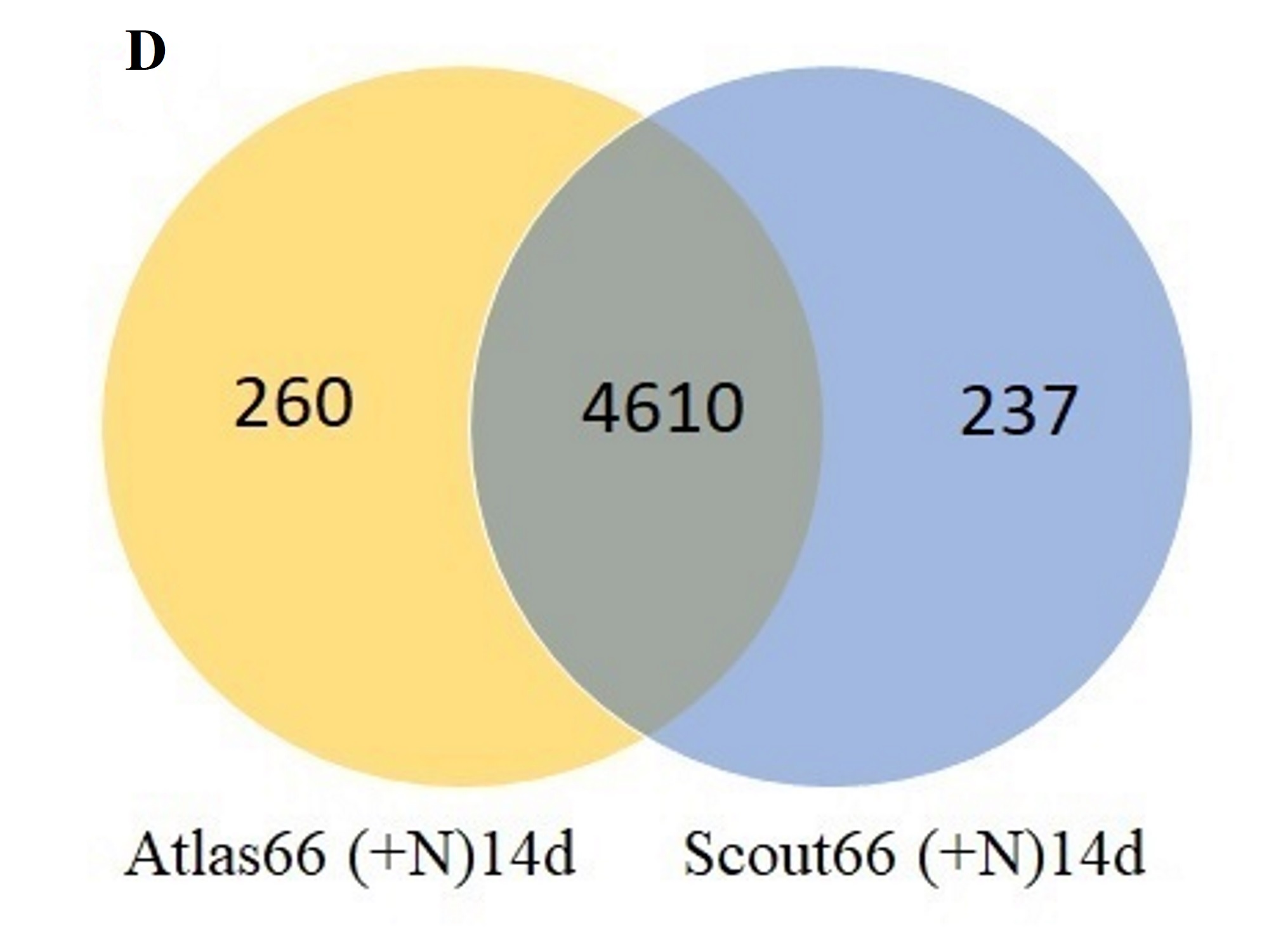

Supplement: Supplementary file 1 [file ijms-21-02119-s001.zip › 新建文件夹 (2)/Supplementary Figure_revised (round 2)/Supplementary Figure S3D.jpg]

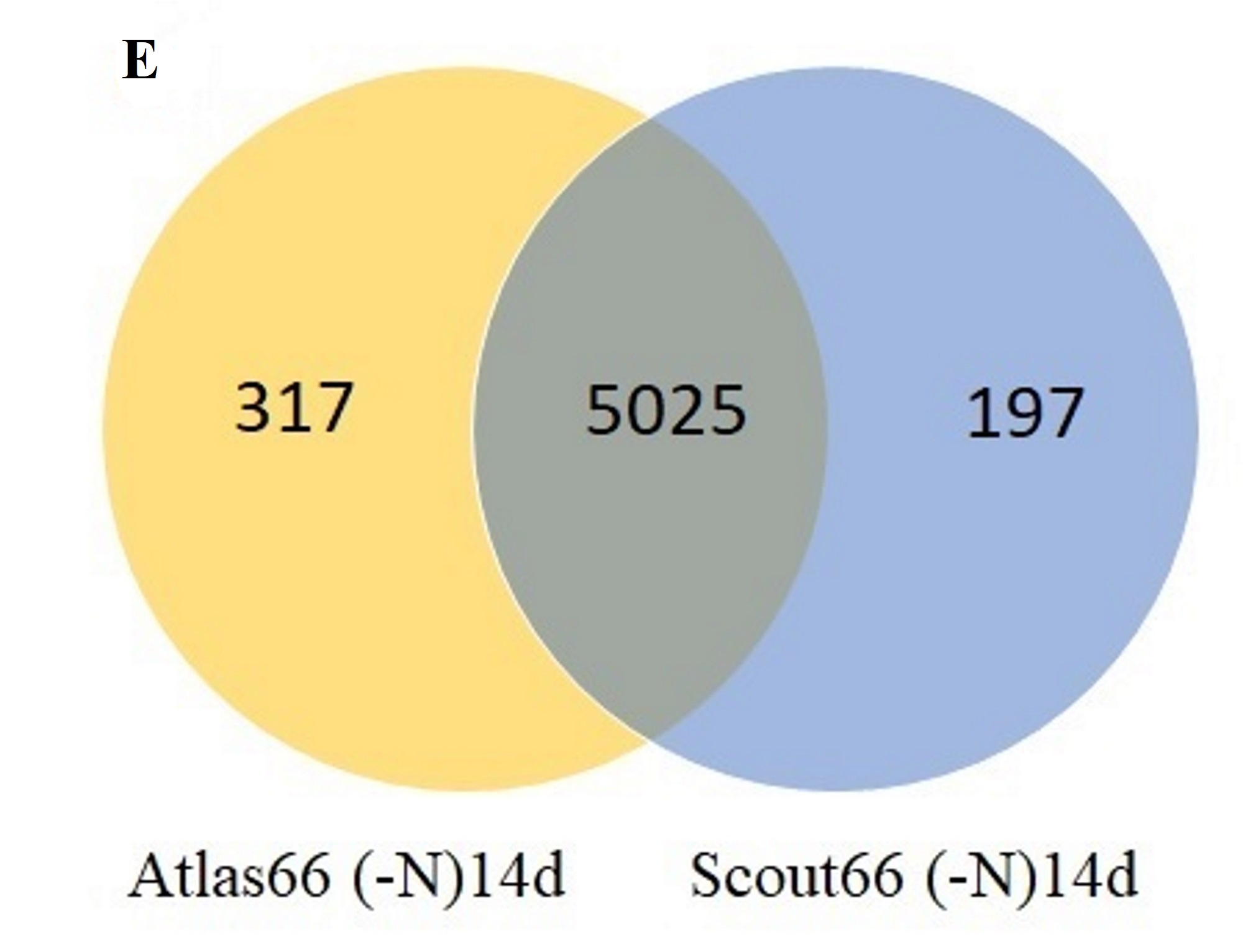

Supplement: Supplementary file 1 [file ijms-21-02119-s001.zip › 新建文件夹 (2)/Supplementary Figure_revised (round 2)/Supplementary Figure S3E.jpg]

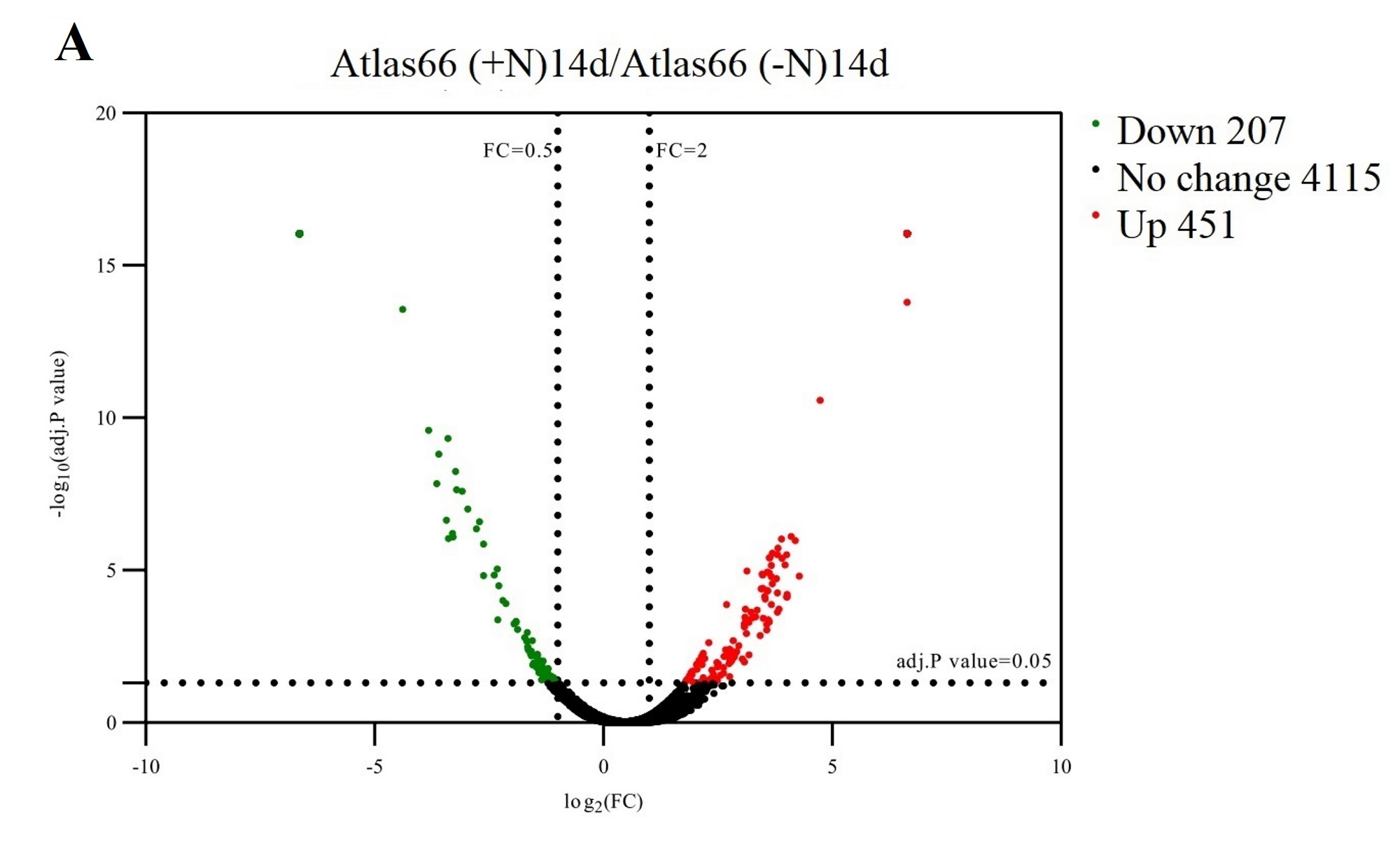

Supplement: Supplementary file 1 [file ijms-21-02119-s001.zip › 新建文件夹 (2)/Supplementary Figure_revised (round 2)/Supplementary Figure S4A.jpg]

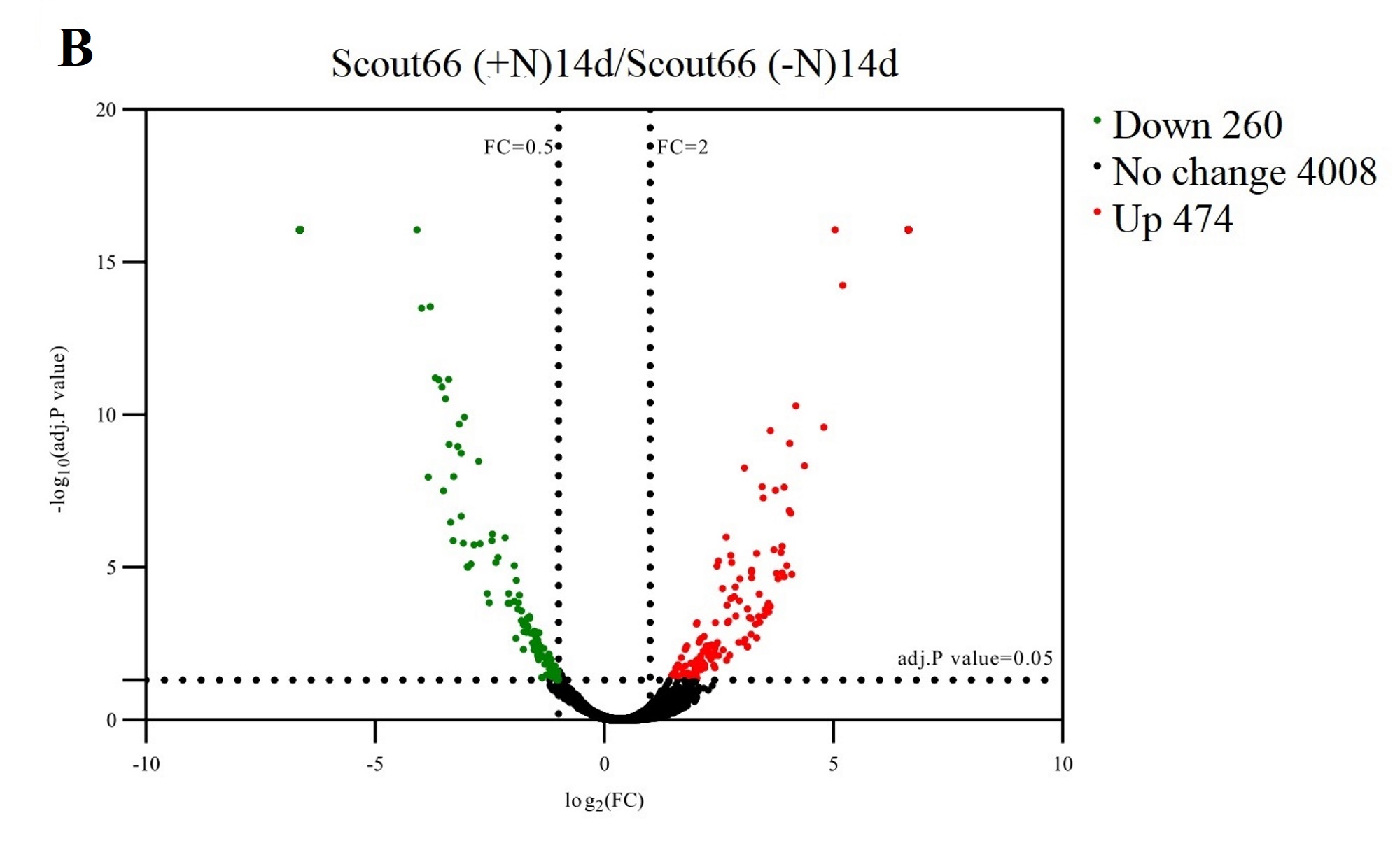

Supplement: Supplementary file 1 [file ijms-21-02119-s001.zip › 新建文件夹 (2)/Supplementary Figure_revised (round 2)/Supplementary Figure S4B.jpg]

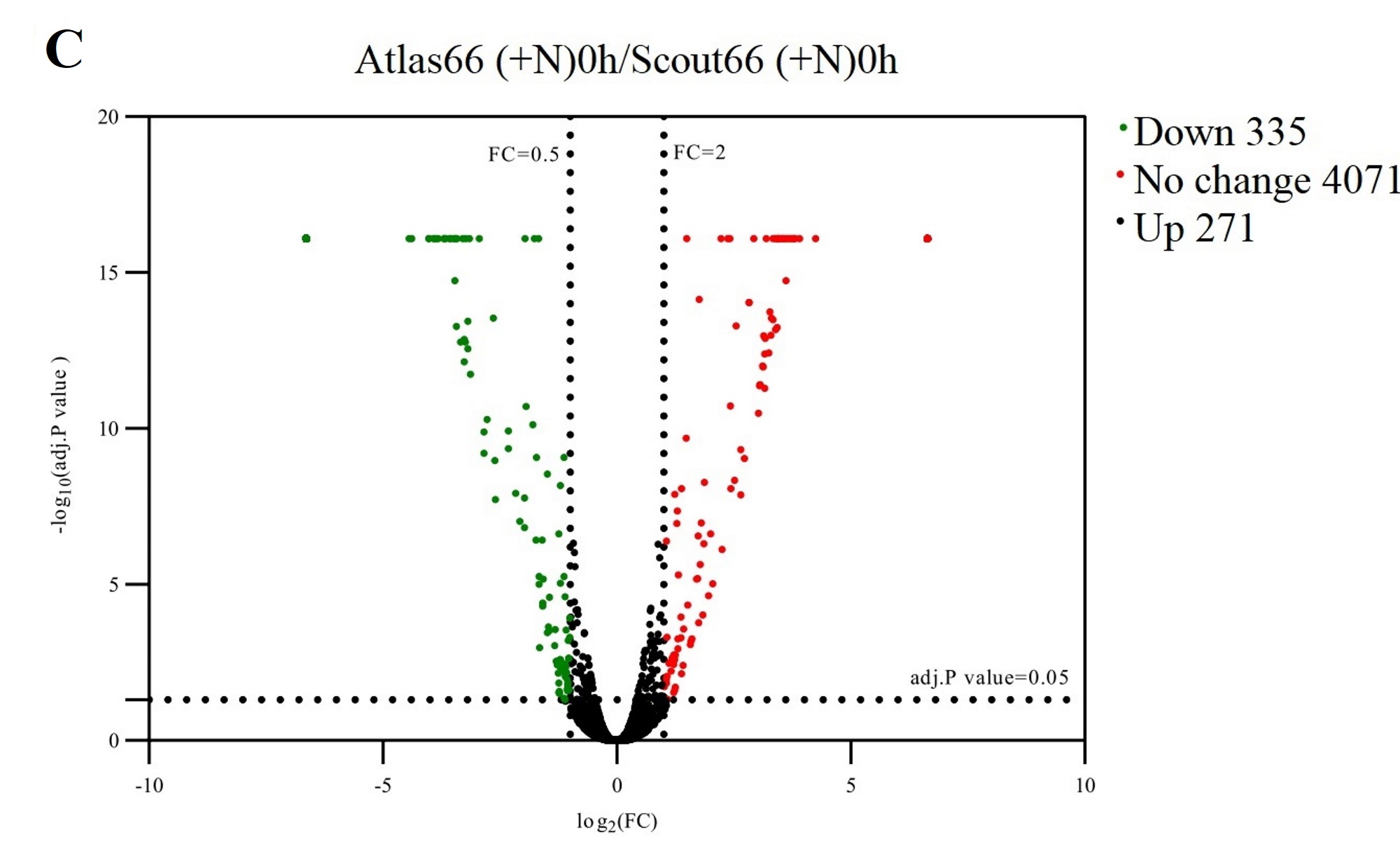

Supplement: Supplementary file 1 [file ijms-21-02119-s001.zip › 新建文件夹 (2)/Supplementary Figure_revised (round 2)/Supplementary Figure S4C.jpg]

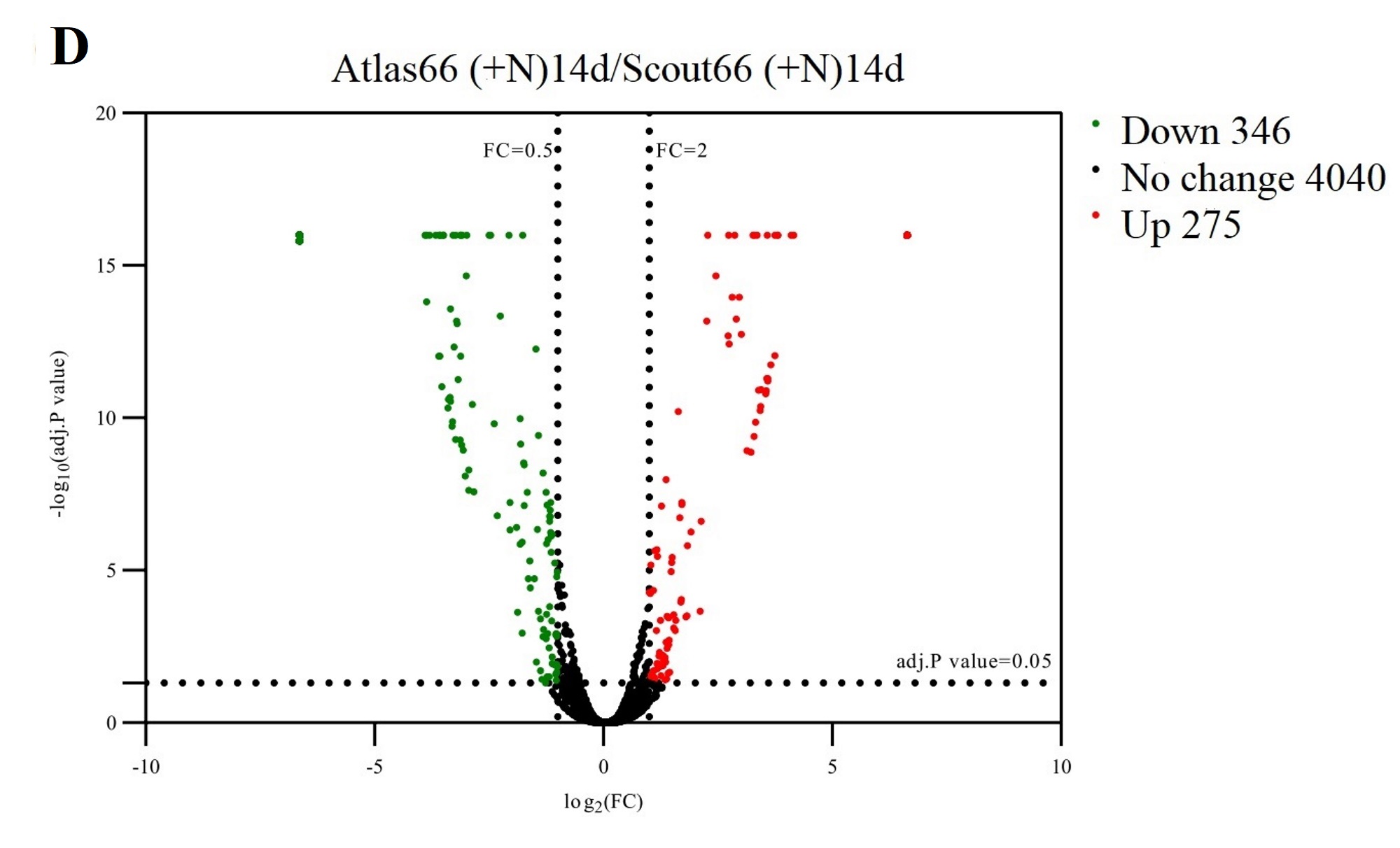

Supplement: Supplementary file 1 [file ijms-21-02119-s001.zip › 新建文件夹 (2)/Supplementary Figure_revised (round 2)/Supplementary Figure S4D.jpg]

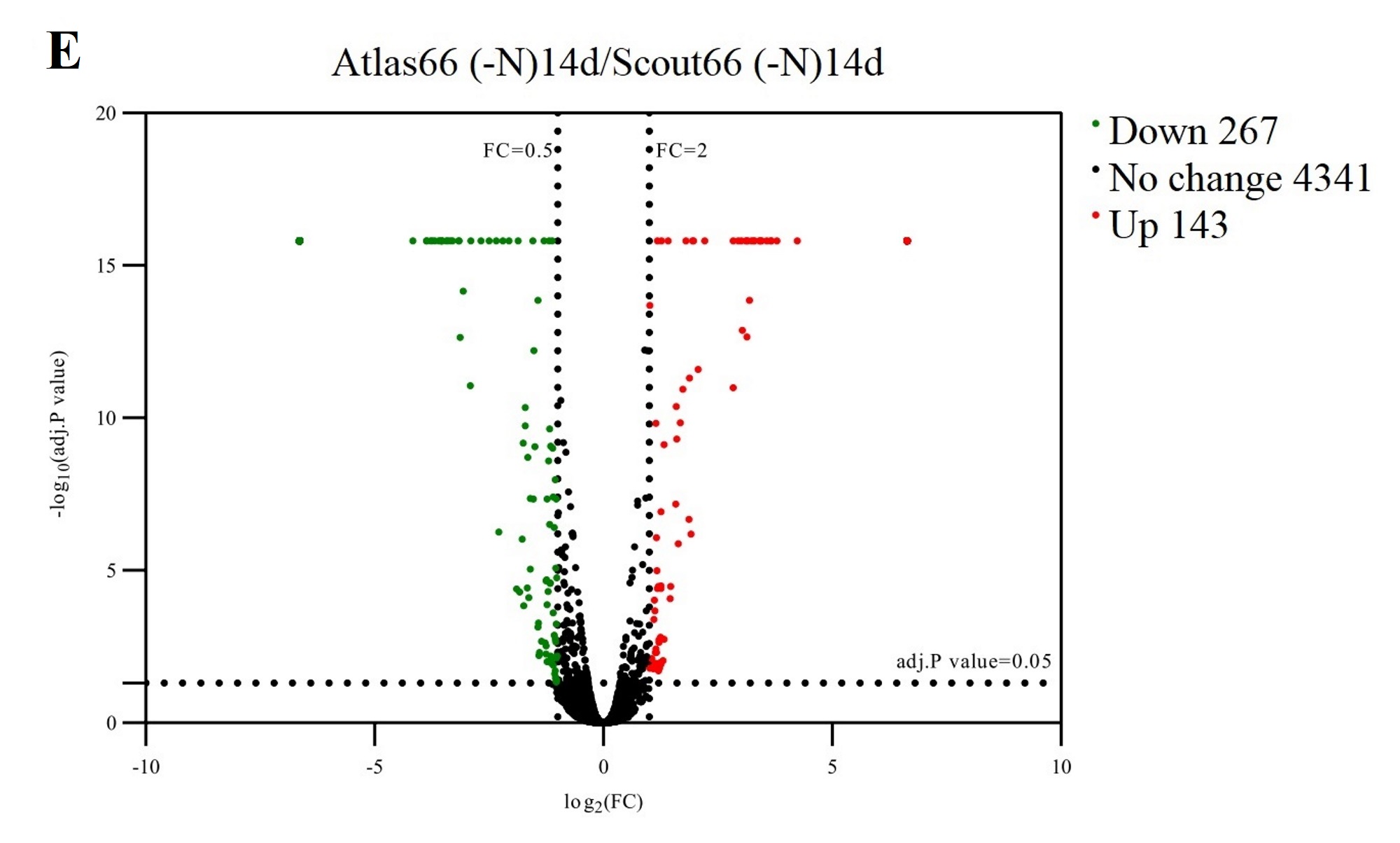

Supplement: Supplementary file 1 [file ijms-21-02119-s001.zip › 新建文件夹 (2)/Supplementary Figure_revised (round 2)/Supplementary Figure S4E.jpg]

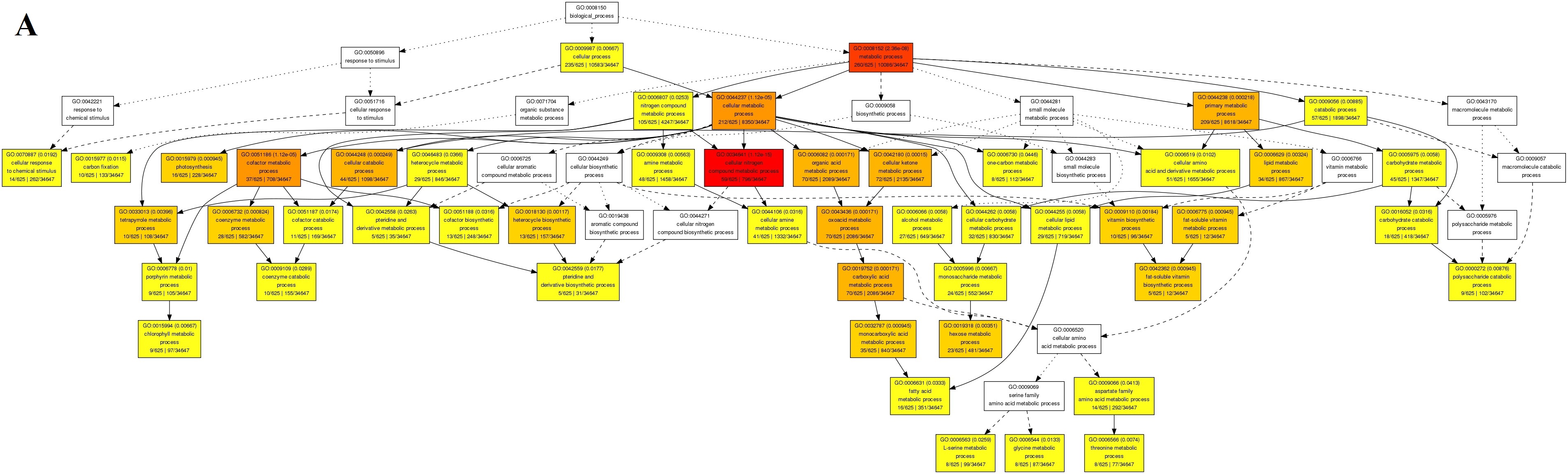

Supplement: Supplementary file 1 [file ijms-21-02119-s001.zip › 新建文件夹 (2)/Supplementary Figure_revised (round 2)/Supplementary Figure S5A.jpg]

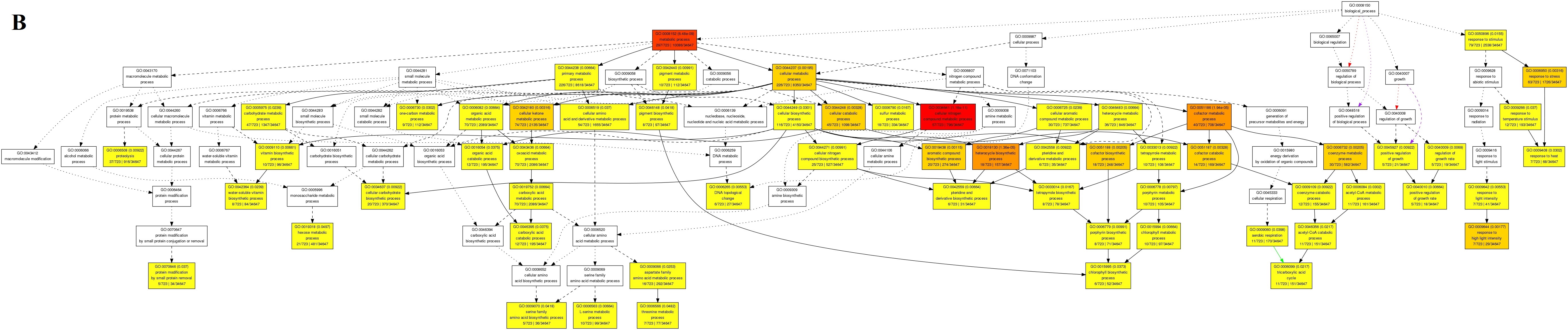

Supplement: Supplementary file 1 [file ijms-21-02119-s001.zip › 新建文件夹 (2)/Supplementary Figure_revised (round 2)/Supplementary Figure S5B.jpg]

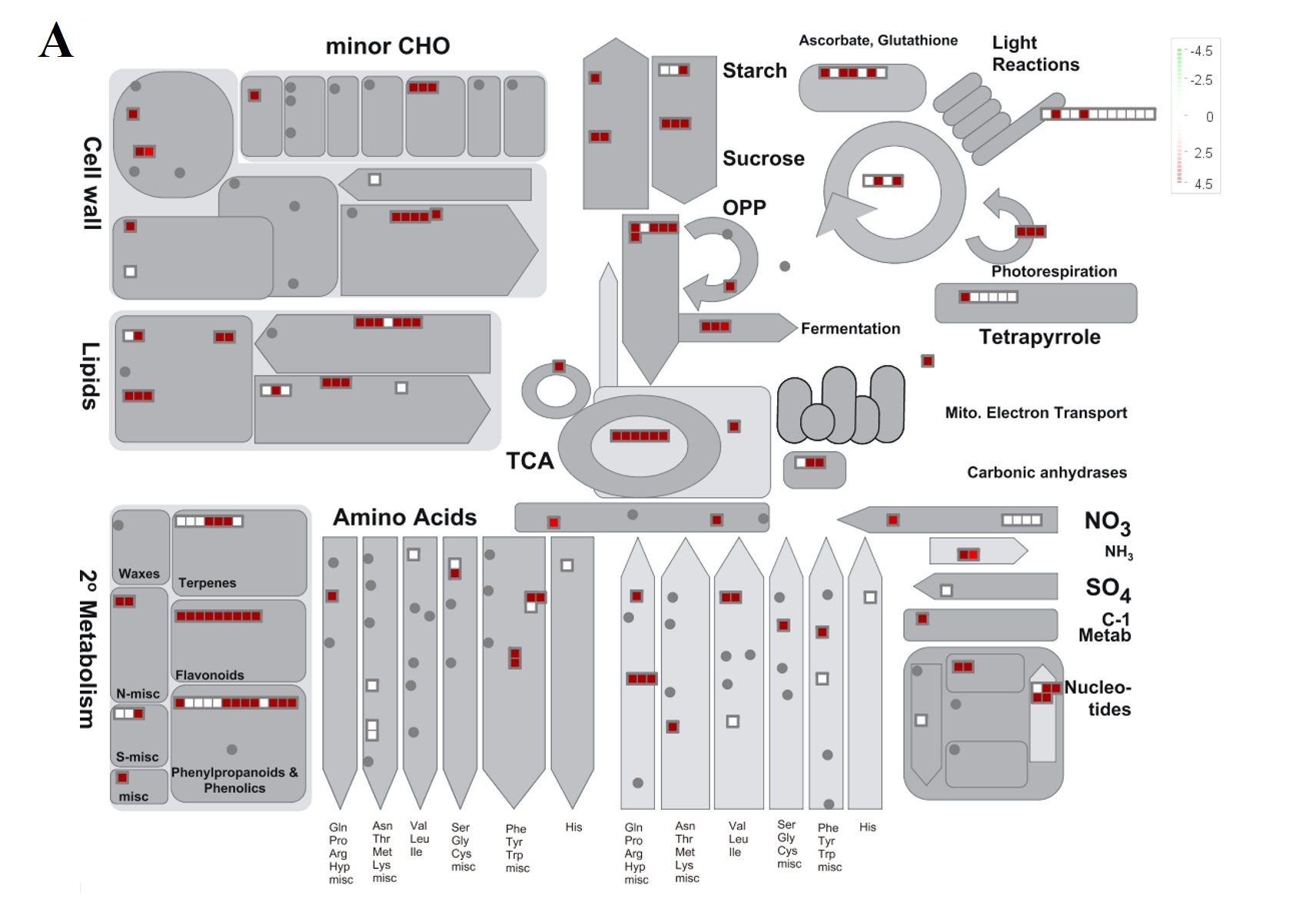

Supplement: Supplementary file 1 [file ijms-21-02119-s001.zip › 新建文件夹 (2)/Supplementary Figure_revised (round 2)/Supplementary Figure S6A.jpg]

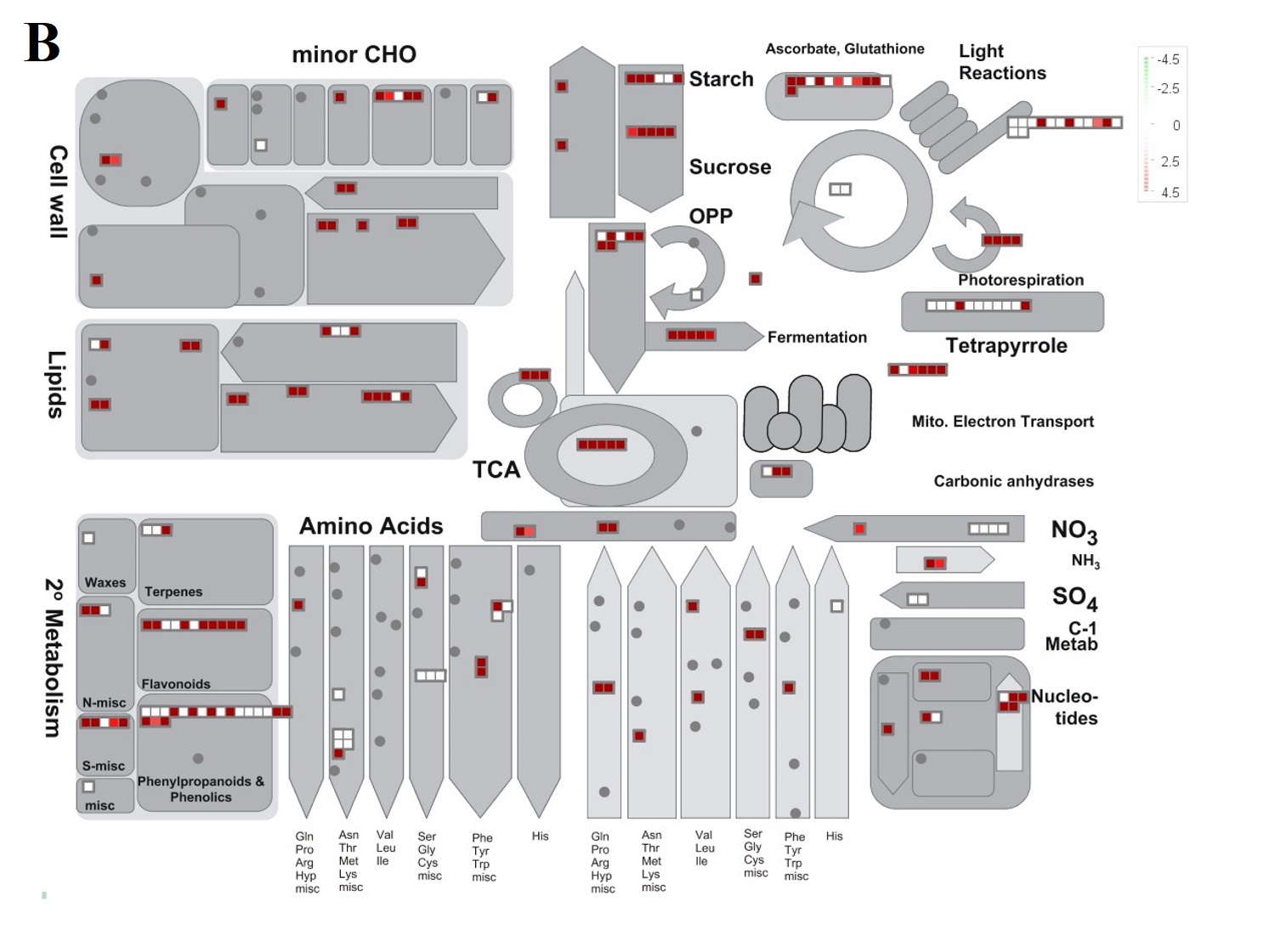

Supplement: Supplementary file 1 [file ijms-21-02119-s001.zip › 新建文件夹 (2)/Supplementary Figure_revised (round 2)/Supplementary Figure S6B.jpg]
